# Engineered probiotic *Bifidobacterium* for tumor-targeted pancreatic cancer therapy

**DOI:** 10.1126/sciadv.adz1388

**Published:** 2026-07-23

**Authors:** Jaehyun Lee, Kaiting Yang, Christina A. Nowicki, Wei Liu, Kangdi Li, Emile Naccasha, Zhichen Sun, Yang-Xin Fu, Hua Liang, Ralph R. Weichselbaum, Mark Mimee

**Affiliations:** ^1^ Department of Microbiology, University of Chicago, Chicago, IL 60637, USA.; ^2^ Department of Radiation and Cellular Oncology, University of Chicago, Chicago, IL 60637, USA.; ^3^ The Ludwig Center for Metastasis Research, University of Chicago, Chicago, IL 60637, USA.; ^4^ Department of Pathology, University of Texas Southwestern, Dallas, TX 75390, USA.; ^5^ Department of Basic Medical Sciences, School of Medicine, Tsinghua University, Beijing 100084, P.R. China.; ^6^ Pritzker School of Molecular Engineering, University of Chicago, Chicago, IL 60637, USA.

## Abstract

Pancreatic ductal adenocarcinoma (PDAC) presents a substantial challenge due to its resistance to cancer treatments. This limited efficacy is, in part, attributed to the immunosuppressive tumor microenvironment (TME), which impairs effector T (T_eff_) cell activity. Interleukin-2 (IL-2) is a key cytokine for T cell activation, but its therapeutic use is limited by a short half-life, systemic toxicity, and regulatory T (T_reg_) activation. To address this limitation, we engineered *Bifidobacterium longum*, a probiotic obligate anaerobe that selectively colonizes the TME, to continuously secrete Super-mutant IL-2 (SumIL-2), an engineered IL-2 variant that preferentially activates T_eff_ cells over T_reg_ cells, thereby delivering SumIL-2 selectively to the tumor (BifidoSumIL-2). Systemic administration of BifidoSumIL-2 significantly suppressed tumor growth in both subcutaneous tumors and orthotopic PDAC in mice, inducing an improved T_eff_/T_reg_ ratio. Combining BifidoSumIL-2 with chemotherapy, radiation, and immunotherapy further restrained orthotopic PDAC growth, highlighting its therapeutic potential for difficult-to-treat cancers like PDAC.

## Teaser

Engineered probiotic *Bifidobacterium* secretes effector T cell–boosting IL-2 variant to reduce pancreatic tumor growth in mice.

## INTRODUCTION

Pancreatic ductal adenocarcinoma (PDAC), which constitutes ∼90% of pancreatic cancer, is a highly aggressive and lethal malignancy (*1*, *2*). Surgical resection, the standard of care, is applicable to only 15 to 20% of patients at diagnosis (*3*). More recently, adjuvant chemotherapy has improved survival for patients undergoing complete resection, but long-term survival in patients who develop metastasis is grave (*1*, *4*, *5*). The tumor microenvironment (TME) in PDAC is highly immunosuppressive, characterized by an abundance of myeloid-derived suppressor cells (*6*, *7*), a deficiency of functional dendritic cells (DCs) (*8*), limited infiltration of effector T (T_eff_) cells (*9*, *10*), an increase in regulatory T (T_reg_) cells (*11*), and severe hypoxia (*12*). These conditions are also observed in the TME of other difficult-to-treat cancers, such as non–small cell lung cancer (*13*), ovarian cancer (*14*), prostate cancer (*15*), and colorectal cancer (*16*), which significantly limit the efficacy of conventional therapies, such as chemotherapy, radiotherapy, and immunotherapy (*1*). Therefore, there is a critical need for innovative therapeutic approaches capable of reshaping the immunosuppressive TME toward a more immunoactive state.

Interleukin-2 (IL-2), a crucial cytokine for T cell proliferation and activation (*17*, *18*), has been used as a cancer immunotherapy to enhance T_eff_ activity in the TME (*19*). Despite its promise, a significant limitation of IL-2 therapy is its concomitant promotion of T_reg_ expansion that can suppress T_eff_ activity within the TME and impede antitumor responses (*20*–*22*). Previously, Super-mutant IL-2 (SumIL-2), an engineered human IL-2 (hIL-2) variant with six amino acid mutations (F42A, L80F, R81D, L85V, I86V, and I92F), was developed to exhibit preferential binding to IL-2 receptor β (IL-2Rβ, also known as CD122) over IL-2Rα (CD25), resulting in selective T_eff_ activation and minimized T_reg_ activation (*23*). SumIL-2 thus effectively converts the immunosuppressive TME to an immunoactive state. However, despite these improvements, IL-2 treatment, including SumIL-2, still suffers from a short in vivo half-life and systemic toxicity (*24*–*26*), highlighting the need for tumor-targeted delivery approaches.

Engineered bacteria have emerged as promising vehicles for tumor-targeted therapeutic delivery (*27*, *28*). *Bifidobacterium* spp., a commensal probiotic obligate anaerobe found in the mammalian gastrointestinal tract (*29*), offer an alternative to other extensively studied bacteria such as *Escherichia coli*, *Salmonella* Typhimurium, and *Listeria monocytogenes* for tumor-targeted therapeutic delivery (*30*, *31*). Due to their probiotic obligate anaerobic nature, *Bifidobacterium* spp. are generally considered safe (*30*) and exhibit a greater propensity to specifically colonize the hypoxic TME without requiring engineered attenuation for safety (*32*–*34*) or metabolite auxotrophy (*33*, *35*) for tumor-specific targeting. Gastrointestinal *Bifidobacterium* spp. have been shown to play a key role in the response to immune checkpoint inhibitors in mice [e.g., anti–programmed death ligand 1 (anti–PD-L1) and anti-CD47] (*36*, *37*). Oral administration of *Bifidobacterium* spp. leads to increased antitumor gene expression within DCs and enhanced T cell activation, converting nonresponders into responders to immune checkpoint inhibitors. *Bifidobacterium* spp. that translocate from the gastrointestinal tract to the TME enhance DC antigen presentation and modulate T cell activity, resulting in synergistic effects when combined with immunotherapy (*36*). Furthermore, the production of immunomodulatory metabolites, such as inosine (*38*, *39*), indole-3-lactic acid (*40*, *41*), and cyclic di–adenosine 5′-monophosphate (*36*), by *Bifidobacterium* spp. can contribute to the antitumor effects. These natural antitumor effects of *Bifidobacterium* spp., along with their tumor-specific targeting, offer a significant advantage in their use as a delivery platform for diverse tumor-targeted therapies, ranging from diagnostic and therapeutic nanoparticles (*42*) to gene expression [e.g., tumor antigens (*43*) and prodrug-activating enzymes (*44*)].

**Fig. 1.**
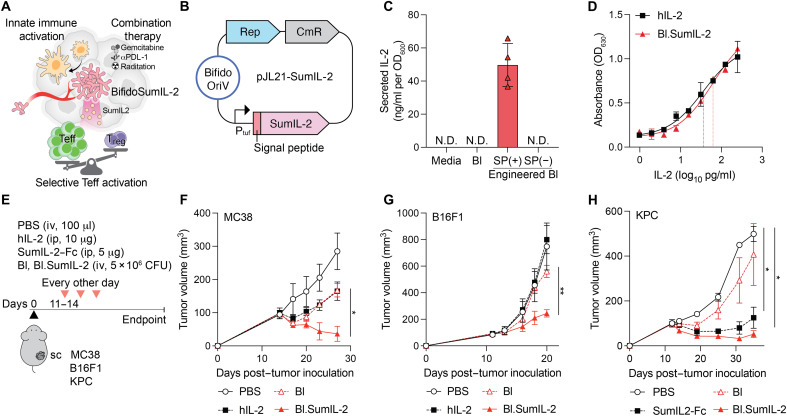
*B. longum* engineered to secrete SumIL-2 suppresses growth of subcutaneous tumors. (**A**) Schematic illustrating the BifidoSumIL-2 platform. BifidoSumIL-2 exerts antitumor effects by activating the innate immune system through a stimulator of interferon genes (STING)–dependent pathway and selectively promoting T_eff_ cell activation over T_reg_ cells. Additionally, BifidoSumIL-2 is combined with chemotherapy, radiation, and immunotherapy to further enhance its antitumor efficacy. (**B**) Plasmid construction for SumIL-2 secretion. *Bifidobacterium* constitutive promoter (P_tuf_) was placed upstream of the SumIL-2 gene, with or without a secretion signal peptide derived from a hypothetical protein (BLON_RS02330) into the pJL21 plasmid. (**C**) The concentration of SumIL-2 (ng per ml) in the culture supernatant was measured using enzyme-linked immunosorbent assay (ELISA) after 8 hours of growth in MRS medium (*n* = 4 per group). N.D., not detected. Limit of detection, 15 pg/ml. (**D**) Bioactivity of recombinant hIL-2 and BifidoSumIL-2 (Bl.SumIL-2) supernatants. Data were fitted to a three-parameter dose-response curve [hIL-2, coefficient of determination (*R*^2^) = 0.9669; BifidoSumIL-2, *R*^2^ = 0. 9860] to determine the median effective concentration (EC_50_) for hIL-2 (36.38 pg/ml; black dotted line) and for BifidoSumIL-2 supernatant (62.19 pg/ml; red dotted line). (**E** to **H**) C57BL/6 mice were subcutaneously injected with 1 × 10^6^ MC38 (F), B16F1 (G), or KPC cells (H) on day 0 and treated with PBS (intravenous, 200 μl), hIL-2 (intraperitoneal, 10 μg), wild-type *B. longum* (Bl; intravenous, 1 × 10^7^ CFU), or BifidoSumIL-2 (Bl.SumIL-2; intravenous, 1 × 10^7^ CFU) on days 14, 16, and 18 (F); days 11, 13, and 15 (G); or days 12, 14, and 16 (H) (*n* = 3 to 4 per group). Mean tumor growth curves for the tumor-bearing mice. Two-way analysis of variance (ANOVA) tests were used to analyze the tumor growth. Data are presented as means ± SEM. *P < 0.05; **P < 0.01. One of the two representative experiments is shown. ip, intraperitoneal; iv, intravenous; sc, subcutaneous.

Here, we engineered *Bifidobacterium longum* to continuously secrete SumIL-2 (BifidoSumIL-2) as a tumor-targeted delivery system that enhances the therapeutic efficacy of SumIL-2 while minimizing systemic toxicity (Fig. 1A). In subcutaneous tumor models, systemically administered BifidoSumIL-2 selectively colonized tumors and exhibited a potent antitumor effect, suppressing tumor growth via a stimulator of interferon genes (STING)– and T cell–dependent mechanism. In an orthotopic PDAC model, BifidoSumIL-2 treatment also suppressed tumor growth by effectively modulating CD8^+^ T cell profiles within the TME. Combining BifidoSumIL-2 with chemotherapy, radiotherapy, or immunotherapy further amplified therapeutic efficacy, highlighting its significant potential for PDAC treatment.

## RESULTS

### Engineered probiotic *B. longum* secreted bioactive SumIL-2 in vitro

SumIL-2 is a modified IL-2 variant designed to selectively activate T_eff_ cells while minimizing T_reg_ cell activation (*23*). *Bifidobacterium* spp. are probiotic bacteria known for their ability to colonize tumors and enhance immunotherapy efficacy (*36*, *37*). We first examined whether *B. longum* ATCC15697 enhances the antitumor efficacy of SumIL-2 (fig. S1). In mice bearing subcutaneous MC38 tumors (murine colorectal carcinoma), intraperitoneal administration of SumIL-2–Fc, a fusion of SumIL-2 with an Fc domain engineered to improve stability and half-life (*23*), resulted in an additive effect when combined with intravenous administration of *B. longum*, compared with either treatment alone. These results suggest that engineering *B. longum* to secrete SumIL-2 could provide a synergistic and cost-effective strategy for sustained intratumoral IL-2 delivery and enhanced antitumor efficacy.

To explore this approach, we cloned the SumIL-2 gene under a constitutive promoter of elongation factor Tu (P_tuf_) into the pJL21 plasmid, either fused to an N-terminal secretion signal peptide derived from the hypothetical secreted protein (BLON_RS02330) or lacking the signal peptide (Fig. 1B). These constructs were transformed into *B. longum*, and SumIL-2 was detected in culture supernatants exclusively in the engineered *B. longum* expressing SumIL-2 fused to the signal peptide, whereas no extracellular SumIL-2 was detected in the wild-type *B. longum* or control strain lacking the signal peptide (Fig. 1C). Hereafter, the SumIL-2–secreting *B. longum* is referred to as BifidoSumIL-2. We next evaluated the bioactivity of the secreted SumIL-2 using human embryonic kidney (HEK)–IL-2 reporter cells (Fig. 1D). The secreted SumIL-2 exhibited similar bioactivity compared with hIL-2, with median effective concentration value of 36 pg/ml for hIL-2 and 62 pg/ml for secreted SumIL-2, suggesting that production and secretion from a bacterial host did not compromise SumIL-2 function.

To assess plasmid stability in the absence of antibiotic selection, we performed an in vitro serial passaging assay under nonselective conditions (fig. S2). BifidoSumIL-2 was passaged daily for 7 consecutive days in the presence or absence of antibiotic selection. No significant difference in colony-forming unit (CFU) counts was observed between antibiotic-free cultures and antibiotic-selected controls, confirming robust plasmid retention over more than 70 generations. Additionally, SumIL-2 secretion was maintained after 7 days without antibiotic selection, indicating its stable and functional expression (fig. S2B).

To evaluate the in vivo efficacy of BifidoSumIL-2 (Fig. 1E), we treated mice bearing established subcutaneous MC38 tumors every 2 days with three intravenous doses of either phosphate-buffered saline (PBS; control), wild-type *B. longum*, or BifidoSumIL-2 or intraperitoneal injections of hIL-2. BifidoSumIL-2 treatment suppressed tumor growth significantly more than the other treatments, demonstrating the additive effect of combining *B. longum* and SumIL-2 (Fig. 1F). To explore the broader therapeutic potential of BifidoSumIL-2, we further conducted similar experiments using mice bearing the subcutaneous B16F1 tumor (murine melanoma), characterized by its immunosuppressive properties due to down-regulation of major histocompatibility complex (MHC) class I and absence of MHC class II expression (*45*). BifidoSumIL-2 treatment significantly suppressed tumor growth compared with the other treatments, pointing to its ability to overcome resistance to treatments with wild-type *B. longum* or hIL-2 alone (Fig. 1G). Additionally, we examined the effect of BifidoSumIL-2 in mice injected subcutaneously with pancreatic tumor cells, primarily isolated from LSL-*Kras*^*G12D/+*^, LSL-*Trp53*^*R172H/+*^, *Pdx-1*-Cre (KPC) mice (Fig. 1H) (*46*). BifidoSumIL-2 treatment led to significantly greater tumor suppression than PBS and *B. longum* treatments while showing no significant difference from SumIL-2–Fc treatment. In a metastatic Lewis lung carcinoma (LLC) model, where LLC cells were intravenously injected to establish lung tumors, BifidoSumIL-2 reduced both the number and size of lung nodules (fig. S3). Collectively, these results confirmed the robust antitumor efficacy of systemically administered BifidoSumIL-2 across multiple tumor models.

To assess tissue biodistribution after systemic administration, we collected and analyzed tumor and organ samples from mice with subcutaneous B16F1 tumors at various time points after a single intravenous injection of BifidoSumIL-2 (fig. S4A). From day 1 through day 7, BifidoSumIL-2 was consistently and exclusively detected in the tumors, with no detection in the blood, liver, spleen, kidney, heart, or lung, indicating its selective tumor colonization (fig. S4, B and C). To further evaluate clearance of BifidoSumIL-2, we extended the monitoring of tumor bacterial loads through day 13 postadministration. We observed a progressive decline in intratumoral bacterial load over time. By day 10, one of the three tumors showed complete clearance, and, by day 13, no colonies were detectable in any tumors examined, confirming that BifidoSumIL-2 was effectively cleared by the host (fig. S4B). Furthermore, by measuring the concentration of IL-2 in tumors via enzyme-linked immunosorbent assay (ELISA), we observed prolonged IL-2 levels in tumors (fig. S4D). We note that human and murine IL-2 exhibit cross-reactivity; therefore, the measured IL-2 levels represented the combined level of both the secreted SumIL-2 by BifidoSumIL-2 and endogenous mouse IL-2.

We also examined the safety of BifidoSumIL-2 by monitoring body weight and systemic inflammatory responses following three intravenous administrations in mice bearing B16F1 tumors. BifidoSumIL-2 did not induce critical body weight loss as body weights remained above 95% of baseline throughout the experimental period (Fig. 1G and fig. S5A). Additionally, we assessed potential toxicity by analyzing serum collected 7 days after a single administration to tumor-free mice (fig. S5, B to E). We first measured serum levels of aspartate transaminase (AST) and alanine transaminase (ALT) to evaluate potential hepatic injury (*47*). Whereas ALT levels were significantly elevated by the wild-type *B. longum*, BifidoSumIL-2, and hIL-2 treatment compared with the PBS treatment, AST elevations induced by wild-type *B. longum* and BifidoSumIL-2 treatment were significantly lower than those observed with hIL-2 treatment. The AST/ALT ratio showed no significant differences between the PBS, wild-type *B. longum*, and BifidoSumIL-2–treated groups, whereas hIL-2 treatment caused a significant increase, suggesting that BifidoSumIL-2 exhibits reduced hepatic injury compared with hIL-2. This indicates that the targeted delivery of SumIL-2 by BifidoSumIL-2 does not induce the hepatic injury seen with hIL-2. We next examined inflammatory cytokines in serum (fig. S5, F to H) and observed no significant increases in the levels of interferon-γ (IFN-γ), tumor necrosis factor–α (TNFα), and IL-6 in any treatment group (hIL-2, wild-type *B. longum*, or BifidoSumIL-2) compared with the PBS control group. Consistently, analysis of serum collected 7 days after three intravenous administrations in mice bearing B16F1 tumors also revealed no significant increases in these cytokines in any treatment group compared with PBS controls (fig. S5, I to L). Together, these results demonstrate that BifidoSumIL-2 exhibits robust antitumor efficacy through a combined effect of the *B. longum* chassis and SumIL-2 activity, selectively targeting tumors and maintaining a favorable safety profile.

### BifidoSumIL-2 elicits STING- and T cell–dependent antitumor immunity

We next investigated the underlying mechanisms responsible for the synergistic antitumor effects of BifidoSumIL-2. Previous studies have demonstrated that *Bifidobacterium* spp. activate innate immune response through STING signaling pathway, thereby overcoming resistance to immunotherapy (*36*). Additionally, SumIL-2 preferentially binds IL-2Rβ over IL-2Rα, leading to selective activation of T_eff_ cells and minimal activation of T_reg_ cells (*23*). Therefore, we hypothesized that the combined action of *B. longum*–mediated innate immune activation and the secreted SumIL-2 would enhance antitumor immunity by driving T_eff_ cell priming and expansion.

**Fig. 2.**
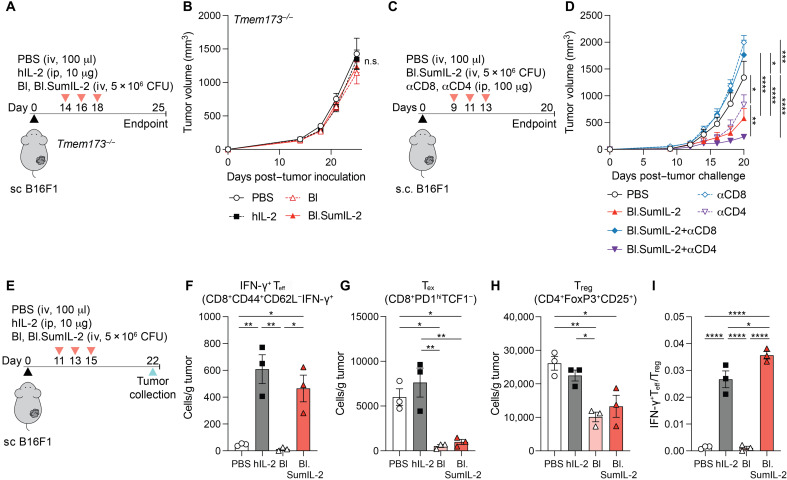
BifidoSumIL-2 antitumor effect depends on STING activation and leads to modulation of T cells in the TME. (**A** and **B**) *Tmem173^−/−^* mice were subcutaneously injected with 1 × 10^6^ B16F1 cells on day 0 and treated with PBS (intravenous, 100 μl), hIL-2 (intraperitoneal, 10 μg), wild-type *B. longum* (Bl; intravenous, 5 × 10^6^ CFU), or BifidoSumIL-2 (Bl.SumIL-2; intravenous, 5 × 10^6^ CFU) on days 14, 16, and 18 (*n* = 4 to 5 per group). (**C** and **D**) C57BL/6 mice were subcutaneously injected with 1 × 10^6^ of B16F1 cells on day 0 and treated with PBS (intravenous, 100 μl), BifidoSumIL-2 (Bl.SumIL-2; intravenous, 5 × 10^6^ CFU), anti-CD8 antibody (αCD8; intraperitoneal, 100 μg), or anti-CD4 antibody (αCD4; intraperitoneal, 100 μg) on days 9, 11, and 13 (*n* = 5 per group). [(B) and (D)] Mean tumor growth curves for the tumor-bearing mice. Two-way ANOVA was used to analyze the tumor growth data. Only selected statistically significant differences are shown. (**E** to **I**) C57BL/6 mice were subcutaneously injected with 1 × 10^6^ of B16F1 cells on day 0 and treated with PBS (intravenous, 100 μl), hIL-2 (intraperitoneal, 10 μg), wild-type *B. longum* (Bl; intravenous, 5 × 10^6^ CFU), or BifidoSumIL-2 (Bl.SumIL-2; intravenous, 5 × 10^6^ CFU) on days 11, 13, and 15. Tumor immune cell populations were analyzed by flow cytometry 7 days after the final treatment (*n* = 3 per group). [(F) and (H)] T cell subsets were gated on CD45^+^CD3^+^ cells in the TME. Gating strategy and representative dot plots are shown in fig. S7. The number of IFN-γ^+^ T_eff_ (CD8^+^CD44^+^CD62L^−^) (F), T_ex_ (PD-1^+^TCF1^−^ CD8^+^) (G), and T_reg_ (FoxP3^+^CD25^+^ CD4^+^) (H) cells normalized to tumor weight (cells per gram). (I) The ratio of IFN-γ^+^T_eff_ cells to T_reg_ cells in the TME. (F to I) One-way ANOVA was used to compare groups. Data are presented as means ± SEM. n.s., not significant; *P < 0.05; **P < 0.01; ***P < 0.001; ****P < 0.0001. One of the two representative experiments is shown. ip, intraperitoneal; iv, intravenous; sc, subcutaneous.

To test this hypothesis, we first cocultured bone marrow–derived myeloid cells (BMDMs) and B16F1 cells in vitro in the presence of PBS control, wild-type *B. longum*, or BifidoSumIL-2 and then isolated BMDMs for further analysis. BifidoSumIL-2–treated group exhibited increased phosphorylation of interferon regulatory factor 3 (pIRF3), a key transcription factor downstream of STING activation (fig. S6A) (*48*, *49*). Consistent with this, BifidoSumIL-2 treatment up-regulated IRF3-dependent genes, such as *Ifn*β, *Cxcl10*, and *Isg15* (*50*) compared with PBS control and wild-type *B. longum* treatment (fig. S6, B to D). These increases were abolished when BMDMs derived from STING-deficient mice were used, confirming that activation was STING dependent (fig. S6, A to D). We also conducted experiments using STING-deficient (*Tmem173^−/−^*) mice. BifidoSumIL-2 treatment failed to elicit an antitumor effect in *Tmem173^−/−^* mice bearing the subcutaneous B16F1 tumor, indicating that STING signaling is required for therapeutic efficacy (Fig. 2, A and B). Because pathogen-associated molecular patterns derived from BifidoSumIL-2 may activate innate immune responses by binding Toll-like receptors and initiating MyD88-dependent signaling, we investigated whether MyD88 contributed to the antitumor effects (fig. S6, E and F). However, MyD88-deficiency did not abrogate the antitumor effects of BifidoSumIL-2. Together, we demonstrated that antitumor efficacy of BifidoSumIL-2 is STING-dependent, but MyD88-independent.

Furthermore, we evaluated the contribution of adaptive immunity using adaptive immunity-deficient (*Rag1^−/−^*) mice bearing subcutaneous B16F1 tumors, in which the therapeutic efficacy of BifidoSumIL-2 was completely lost (fig. S6, G and H). To identify the specific T cell subset responsible for this response, we performed in vivo antibody-mediated depletion (Fig. 2, C and D). The efficacy of BifidoSumIL-2 was abrogated upon CD8^+^ T cell depletion, whereas CD4^+^ T cell depletion did not impair its efficacy, indicating that its therapeutic activity is CD8^+^ T cell dependent. Collectively, these results demonstrate that the antitumor effect of BifidoSumIL-2 relies on both innate STING activation and CD8^+^ T cell–mediated adaptive immunity.

To evaluate immune response within the TME induced by BifidoSumIL-2, we harvested subcutaneous B16F1 tumors 7 days after the final treatment and first profiled T cells via flow cytometry (Fig. 2E and fig. S7A). Compared with PBS treatment, BifidoSumIL-2 treatment significantly increased the numbers of IFN-γ^+^ T_eff_ cells (CD8^+^CD44^+^CD62L^−^IFN-γ^+^) and reduced the numbers of exhausted T (T_ex_) cells (CD8^+^PD-1^hi^TCF1^−^) and T_reg_ cells (CD4^+^FoxP3^+^CD25^+^) (Fig. 2, F to H, and fig. S7, B to D). In contrast, hIL-2 treatment increased the numbers of IFN-γ^+^ T_eff_ cells, T_reg_ cells, and T_ex_ cells, whereas *B. longum* treatment reduced both numbers of T_reg_ cells and T_ex_ cells but did not increase the number of IFN-γ^+^ T_eff_ cells. Additionally, BifidoSumIL-2 treatment induced a significantly higher ratio of IFN-γ^+^ T_eff_/T_reg_ than other groups, indicating that BifidoSumIL-2 induced a more favorable immune environment for T_eff_ activity than other treatments (Fig. 2I). In a further analysis of tumor-infiltrating immune cells, BifidoSumIL-2 treatment led to a significant increase in the infiltration of DCs (CD11c^+^MHCII^+^), natural killer (NK) cells (CD3^−^NK1.1^+^), and macrophages (CD11b^+^F4/80^+^) compared with the PBS control, whereas no significant differences were observed in neutrophils (CD11b^+^Ly6G^+^) and monocytes (CD11b^+^Ly6G^−^Ly6C^+^) (figs. S8 and S9). Notably, DC infiltration was significantly higher in the BifidoSumIL-2–treated group than in the hIL-2–treated group. Furthermore, BifidoSumIL-2 treatment reshaped the cytokine profile of TME toward a more proinflammatory antitumor state, increasing the levels of IFN-γ and TNFα (fig. S10) (*51*). Last, splenic T cell populations showed a significant increase in IFN-γ^+^ CD8 T cells and a decrease in T_reg_ cells, along with an elevated CD8^+^IFN-γ^+^/T_reg_ ratio, in the BifidoSumIL-2–treated group. These findings suggest that CD8^+^ T cell effector function is systemically enhanced and not restricted to the TME (fig. S11). Together, these findings demonstrate that BifidoSumIL-2 enhanced CD8^+^ T cell responses and infiltration both locally within the tumor and systemically to promote a favorable antitumor response.

### BifidoSumIL-2 reduces orthotopic PDAC burden and reshapes the TME

**Fig. 3.**
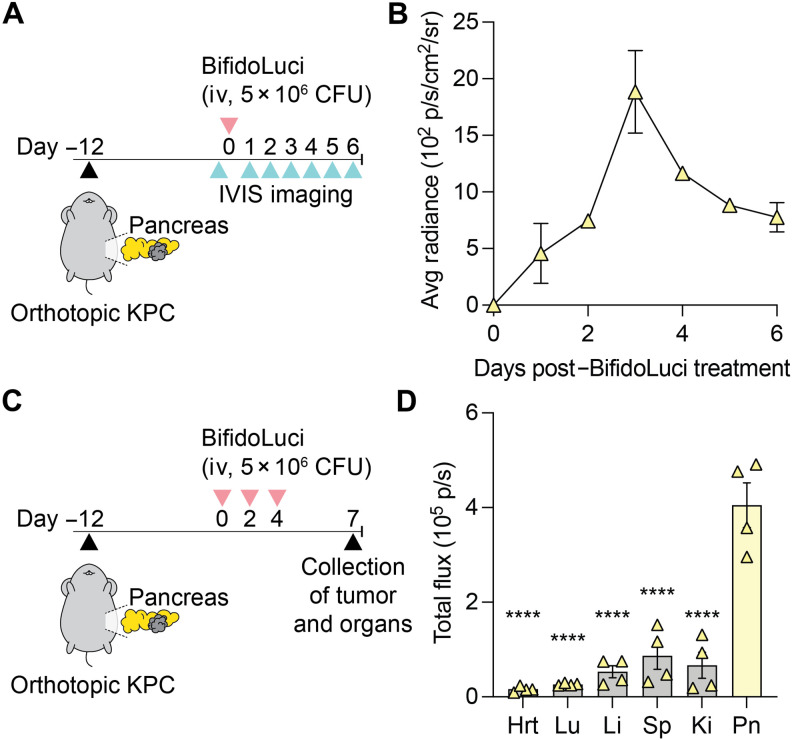
Engineered *B. longum* selectively colonizes orthotopic PDAC tumors. (**A** and **B**) *B. longum* engineered to secrete NanoLuc luciferase (BifidoLuci) was administered once (intravenous, 5 × 10^6^ CFU) to mice bearing orthotopic KPC tumors. (B) Bioluminescence was monitored daily for 6 days post–BifidoLuci treatment (*n* = 2 per group). One experiment is shown. (**C** and **D**) BifidoLuci was administered (intravenous, 5 × 10^6^ CFU) to mice bearing orthotopic KPC tumors three times. Organs and KPC tumors were collected 3 days after the final treatment. (D) Total flux values were shown in photons per second (p/s) (*n* = 4 per group). Hrt, heart; Lu, lung; Li, liver; Sp, spleen; Ki, kidney; Pn, pancreas. One-way ANOVA was used for statistical analysis. One of the two experiments is shown. Data are presented as means ± SEM. ****P < 0.0001. iv, intravenous.

The poor efficacy of conventional therapies in PDAC is primarily due to its highly immunosuppressive TME, which is marked by deficient DCs, sparse activated T_eff_ cells, and enriched T_reg_ cells (*9*, *10*). Building on findings from subcutaneous tumor models that showed that BifidoSumIL-2 could suppress tumor growth by promoting T_eff_ cell activation and infiltration both within the tumor and systemically, we next established orthotopic PDAC by directly injecting KPC cells into the pancreas to improve the clinical relevance of our study. Because PDAC is known for its poor vascularization (*52*), which may hinder the colonization of intravenously injected *B. longum*, we initially assessed *B. longum* colonization within orthotopic KPC tumors. We administered a luciferase-secreting *B. longum* strain (BifidoLuci), via a single intravenous injection starting 12 days after injecting KPC cells into the pancreas tail (Fig. 3A and fig. S12A). In vivo bioluminescence imaging detected the highest luminescence signals in the abdominal region, peaking on day 3 postinjection and subsequently declining (Fig. 3B and fig. S12B). Additionally, after three intravenous BifidoLuci injections every 2 days, we collected pancreatic tumors and other organs on day 7 post–first injection and measured luminescence signals (Fig. 3C). We detected significantly higher luminescence signals in the pancreatic tumors compared with those in other organs (Fig. 3D and fig. S12C). Therefore, we demonstrated that intravenously injected *B. longum* successfully and selectively colonizes orthotopic KPC tumors.

**Fig. 4.**
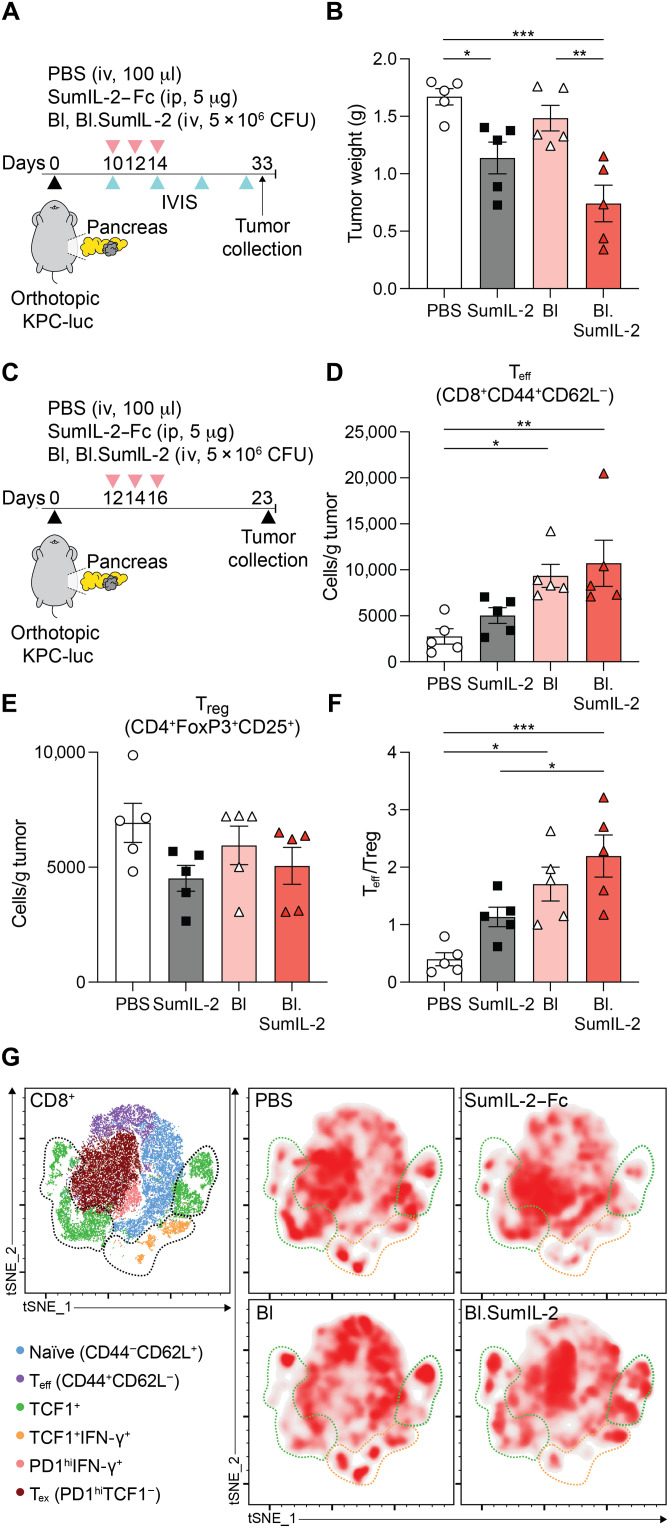
BifidoSumIL-2 reshapes the PDAC TME immune landscape. (**A** and **B**) C57BL/6 mice were injected with 5 × 10^5^ cells of KPC-luc cells into the pancreas tail on day 0 and treated with PBS (intravenous, 100 μl), SumIL-2–Fc (intraperitoneal, 5 μg), wild-type *B. longum* (Bl; intravenous, 5 × 10^6^ CFU), or BifidoSumIL-2 (Bl.SumIL-2; intravenous, 5 × 10^6^ CFU) on days 9, 11, and 13 (*n* = 5 per group). (B) Final tumor weights (days 28 to 33) per treatment group. One-way ANOVA was used to compare groups. Tumors from mice found dead with signs of prolonged postmortem decomposition were excluded from the analysis. (**C** to **G**) C57BL/6 mice were injected with 5 × 10^5^ cells of KPC-luc cells in the pancreas tail on day 0 and treated with PBS (intravenous, 100 μl), SumIL-2–Fc (intraperitoneal, 5 μg), wild-type *B. longum* (Bl; intravenous, 5 × 10^6^ CFU), or BifidoSumIL-2 (Bl.SumIL-2; intravenous, 5 × 10^6^ CFU) on days 12, 14, and 16. Tumor immune cell populations were analyzed by flow cytometry 7 days after the final treatment (day 23, *n* = 5 per group). (D and E) T cell subsets were gated on CD45^+^CD3^+^ cells in the TME. Gating strategy and representative dot plots are shown in fig. S7. The number of T_eff_ (CD8^+^CD44^+^CD62L^−^) (D) and T_reg_ (FoxP3^+^CD25^+^CD4^+^) (E) cells normalized to tumor weight (cells per gram). (F) The ratio of T_eff_ cells to T_reg_ cells in the TME. (G) Uniform manifold approximation and projection (UMAP) clustering of marker expression profiles of 48,000 live CD8^+^ T cells analyzed by flow cytometry. Six distinct T cell clusters were identified according to the expression of TCF1, IFN-γ, PD-1, CD44, and CD62L. t-distributed stochastic neighbor embedding (tSNE) density plots from each treatment group. (B and D to G) One of the two representative experiments is shown. One-way ANOVA was used to compare groups. Data are presented as means ± SEM. *P < 0.05; **P < 0.01; ***P < 0.001. ip, intraperitoneal; iv, intravenous.

To evaluate the ability of BifidoSumIL-2 to suppress PDAC, we intravenously administered BifidoSumIL-2 every 2 days for three total doses, starting 10 days after injecting luciferase-expressing KPC (KPC-luc) cells into the pancreas tail (Fig. 4A). We then monitored body weight and tumor growth by measuring bioluminescence signals from the abdominal region of mice (fig. S13, A to C). Body weight remained stable across all treatment groups relative to baseline (day 9) (fig. S13A). Throughout the experimental period, mice treated with BifidoSumIL-2 consistently exhibited lower bioluminescence signals than those treated with PBS, SumIL-2–Fc, or wild-type *B. longum* (fig. S13, B and C). Tumor collection at the experimental endpoint revealed that tumors from BifidoSumIL-2–treated mice had significantly lower weights than those from mice treated with PBS or wild-type *B. longum* (Fig. 4B and fig. S13D).

Next, we collected PDAC tumors 7 days after the final treatment and analyzed them by flow cytometry to assess the impact of BifidoSumIL-2 on immune cell populations within the PDAC TME (Fig. 4, C to G, and fig. S14). Although the number of live CD45^+^ cells was comparable across all groups, BifidoSumIL-2 treatment significantly increased CD8^+^ T cell infiltration within PDAC TME compared with PBS controls, whereas SumIL-2–Fc showed no significant difference (fig. S14E). Other major immune populations, including CD4^+^ T cells, B cells (NK1.1^−^B220^+^), NK cells, DCs, neutrophils, macrophages, and monocytes, did not differ significantly across all groups. Within the T cell compartment, while the number of T_reg_ was comparable across all groups, both wild-type *B. longum* and BifidoSumIL-2 treatment significantly increased the number of T_eff_ cells within PDAC TME and improved the T_eff_/T_reg_ ratio compared with the PBS control group, supporting a CD8^+^ T cell–dominant remodeling of the TME (Fig. 4, D to F). Additionally, the T_eff_/T_reg_ ratio was significantly higher in the BifidoSumIL-2 group than in the SumIL-2–Fc group, supporting the therapeutic potential of *B. longum*–mediated SumIL-2 delivery. Further high-dimensional profiling of 48,000 live CD8^+^ T cells using spectral flow cytometry demonstrated that BifidoSumIL-2 increased the overall abundance of stem-like (TCF1^+^) CD8^+^ T cells, compared with PBS, SumIL-2–Fc, or *B. longum* treatment, and stem-like IFN-γ^+^ (TCF1^+^IFN-γ^+^) CD8^+^ T cells within PDAC TME, compared with PBS or SumIL-2–Fc treatment (Fig. 4G and fig. S15). These stem-like or stem-like/IFN-γ^+^ CD8^+^ T cells are associated with improved patient outcomes after cancer therapy (*53*, *54*). Together, in an orthotopic PDAC model, engineered *B. longum* successfully colonized in the tumor and BifidoSumIL-2 modulated the TME toward a more immunoactive state by enhancing the IFN-γ^+^ T_eff_/T_reg_ ratio and enriching stem-like CD8^+^ T cells.

### BifidoSumIL-2 enhances antitumor efficacy in combination therapies

**Fig. 5.**
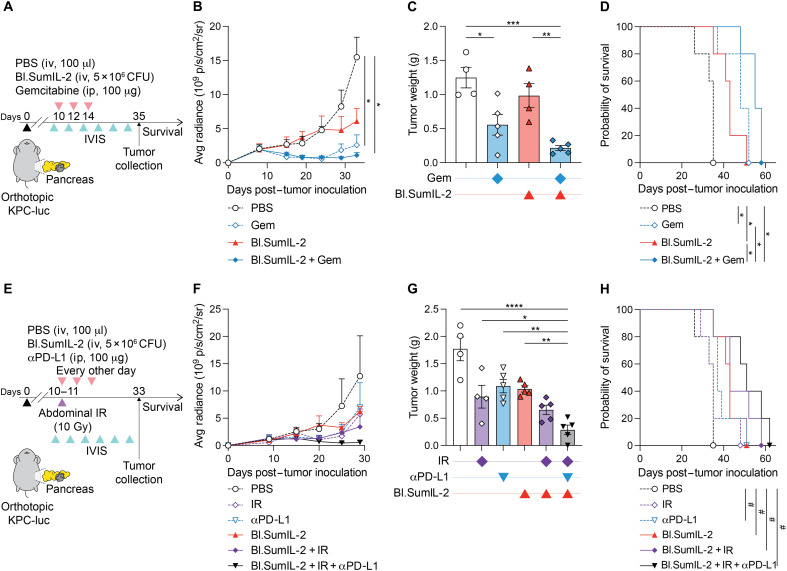
Therapeutic efficacy of BifidoSumIL-2 in combination with chemotherapy, immunotherapy, and abdominal irradiation in an orthotopic PDAC model. (**A** to **D**) C57BL/6 mice were injected with 5 × 10^5^ cells of KPC-luc cells in the pancreas tail on day 0 and were treated with PBS (intravenous, 100 μl), BifidoSumIL-2 (Bl.SumIL-2; intravenous, 5 × 10^6^ CFU), and/or gemcitabine (Gem; intraperitoneal, 100 μg) on days 10, 12, and 14 (*n* = 5 per group). (B) Tumor burden measured by bioluminescence over time. (C) Final tumor weights (days 33 to 35) per treatment group. (D) Kaplan-Meier survival curves were generated up to day 58. Statistical significance was determined using the log-rank (Mantel-Cox) test with multiple comparisons correction. (**E** to **H**) Mice were injected with KPC-luc cells in the pancreas tail on day 0 and were treated with PBS (intravenous, 100 μl), BifidoSumIL-2 (Bl.SumIL-2; intravenous, 5 × 10^6^ CFU), and/or anti–PD-L1 antibody (αPD-L1; intraperitoneal, 100 μg) on days 11, 13, and 15 and/or abdominal irradiation [ionizing radiation (IR); 10 gray (Gy)] on day 11 (*n* = 5 per group). (F) Tumor burden measured by bioluminescence over time. (G) Final tumor weights (days 25 to 33) per treatment group. (H) Mice were treated on days 10, 12, and 14 (*n* = 5 per group). Kaplan-Meier survival curves were generated up to day 62. Statistical significance was determined using the log-rank (Mantel-Cox) test with multiple comparisons correction, with comparisons limited to the PBS and triple-combination groups. [(B) and (F)] Two-way ANOVA was used to analyze the tumor growth data. [(C) and (G)] One-way ANOVA was used to compare groups. Tumors from mice found dead with signs of prolonged postmortem decomposition were excluded from the analysis. [(D) and (H)] These experiments were performed simultaneously; therefore, the PBS control and BifidoSumIL-2 monotherapy groups are shared between the two panels. Data are presented as means ± SEM. *P < 0.05; **P < 0.01; ***P < 0.001; ****P < 0.0001; #*P* = 0.0581. One of the two representative experiments is shown. ip, intraperitoneal; iv, intravenous.

Combination therapies maximize therapeutic benefits while minimizing tumor recurrence or resistance (*55*). We next explored the potential of combining BifidoSumIL-2 with standard cancer therapies such as chemotherapy, radiotherapy, and immune checkpoint blockade. We first examined the therapeutic efficacy of its combination with gemcitabine, a common chemotherapy agent used in various cancers, including PDAC (*1*). BifidoSumIL-2 was administered intravenously, either alone or with intraperitoneal gemcitabine injection, and tumor progression was subsequently monitored using bioluminescence imaging throughout the experimental period (Fig. 5A and fig. S16). Body weight was monitored and remained stable across all treatment groups relative to baseline (day 10) (fig. S16A). Bioluminescence imaging showed significantly greater tumor suppression with gemcitabine alone or in combination with BifidoSumIL-2 compared with PBS (Fig. 5B and fig. S16B). Analysis of tumor weights at the experimental endpoint further confirmed that the combination of BifidoSumIL-2 and gemcitabine resulted in greater tumor growth inhibition compared with PBS, BifidoSumIL-2, or gemcitabine alone (Fig. 5C and fig. S16C). Consistently, the combination of BifidoSumIL-2 and gemcitabine improved survival compared with both the PBS control and the respective monotherapy groups (Fig. 5D).

Additionally, we explored the therapeutic potential of combining BifidoSumIL-2 with ionizing radiation (IR) and immune checkpoint blockade using an anti–PD-L1 antibody. Mice were treated with intravenous BifidoSumIL-2 injections alone or in combination with abdominal IR, with or without intraperitoneal injection of an anti–PD-L1 antibody (Fig. 5E). Throughout the experimental period, all mice treated with the double combination of BifidoSumIL-2 and IR or the triple combination of BifidoSumIL-2, IR, and anti–PD-L1 antibody maintained more than 95% of their baseline body weight (day 9) (fig. S16D). Bioluminescence imaging revealed numerically enhanced tumor growth suppression with the triple-combination treatment compared with PBS control and monotherapies (Fig. 5F and fig. S16E). Consistently, further analysis of tumor weights at the experimental endpoint confirmed that the triple combination resulted in significantly greater tumor growth inhibition compared with PBS control or other monotherapies, demonstrating an additive effect that surpasses the efficacy of individual therapies (Fig. 5F and fig. S16F). In survival analysis, both the double and triple combinations demonstrated prolonged median survival compared with PBS controls. Although these differences did not reach statistical significance (*P* = 0.0581), the survival trend was consistent with the reduced tumor burden at endpoint. To further evaluate the systemic antitumor effect of the triple combination, we established a bilateral subcutaneous KPC model in which localized IR was delivered to one tumor, while BifidoSumIL-2 and anti–PD-L1 were administered systemically (fig. S16). In this setting, the triple combination significantly suppressed growth of the nonirradiated contralateral tumor compared with PBS controls. Collectively, these findings highlight the potential of BifidoSumIL-2 in combination therapies and highlight its role in enhancing treatment efficacy.

## DISCUSSION

Despite recent therapeutic advances, PDAC remains a highly lethal cancer with a poor prognosis due to its immunosuppressive TME (*1*, *2*). In this study, we aimed to achieve tumor targeted drug delivery of SumIL-2, an IL-2 variant designed to selectively activate effector T cells, by using *B. longum*. We engineered *B. longum* to secrete SumIL-2 (BifidoSumIL-2) or luciferase (BifidoLuci) and confirmed selective colonization of engineered *B. longum* in subcutaneous tumor models, as well as an orthotopic PDAC model with KPC cells after systemic administration (Fig. 3 and fig. S4). BifidoSumIL-2 exhibited antitumor effects in subcutaneous tumor models with B16F1, MC38, and KPC cells (Fig. 1, E to H). BifidoSumIL-2 functioned via STING and CD8^+^ T cell–dependent mechanisms (Fig. 2, A to C), leading to increased T_eff_/T_reg_ ratio in both B16F1 flank (Fig. 1, D to H) and orthotopic PDAC models (Fig. 4, F and G). Combining BifidoSumIL-2 with conventional therapies including chemotherapy, radiation, and immunotherapy further enhanced tumor growth inhibition and survival in orthotopic PDAC models (Fig. 5).

Bacteria have gained significant interest as “living drugs” for cancer therapy due to their versatility and ability to thrive in hypoxic TMEs, allowing for targeted drug delivery and minimizing off-target effects (*28*, *30*). Although intravesical Bacillus Calmette-Guérin remains the only US Food and Drug Administration–approved live bacterial therapy for cancer to date, a growing number of clinical studies investigating engineered strains (*56*–*58*), including *E. coli*, *S.* Typhimurium, *L. monocytogenes*, and others, highlight the translational potential of bacteria as programmable therapeutic delivery platforms. Among these, *B. longum* potentially offers a distinct advantage as tumor-targeted delivery platforms, compared with commonly used facultative bacteria such as *E. coli* and *S.* Typhimurium. As an obligate anaerobe, *B. longum* selectively colonizes hypoxic tumor regions without the need for additional strain modification to engineer metabolic auxotrophy or attenuate toxicity (*29*, *30*). Preclinical studies suggest that *Bifidobacterium* spp. can synergize with immunotherapy, likely via STING-mediated innate immune activation within the TME to overcome immune unresponsiveness (*36*, *37*). Additionally, certain *Bifidobacterium* strains produce metabolites such as inosine (*39*) and indole-3-lactic acid (*59*–*61*), which have demonstrated antitumor activity (*38*–*41*). In this study, tumor-colonized BifidoSumIL-2 is expected to exert an additive effect through a dual mechanism: *B. longum*–mediated STING pathway activation of innate immunity and SumIL-2–induced selective T_eff_ cell activation. STING-mediated innate immune activation induces type I interferons and inflammatory cytokines, enhancing antigen presentation to T cells and bridging innate and adaptive immune responses (*62*). Concurrently, SumIL-2 drives robust T_eff_ cells expansion and activation over T_reg_ cells, strengthening adaptive immunity (*23*). As a result, BifidoSumIL-2 treatment preferentially expanded T_eff_ cells over T_reg_ cells within the TME and inhibited tumor growth in both flank and orthotopic PDAC models, effectively shifting the TME toward a more immune-active state. Furthermore, given that most anticancer therapies are administered in combination, including multiple chemotherapies, chemoradiotherapy, or immunotherapy paired with radiotherapy and/or chemotherapy, BifidoSumIL-2 offers a strategy to improve standard cancer treatments. This study demonstrates that BifidoSumIL-2 combined with gemcitabine or radiation and anti–PD-L1 antibodies led to greater tumor growth inhibition than monotherapies alone, underscoring its potential in cancer therapy.

In summary, we present a proof-of-concept for a live bacterial immunotherapy approach using engineered *B. longum*–secreting SumIL-2 to treat the challenging tumor PDAC. Our results suggest that *Bifidobacterium*-mediated delivery of therapeutic proteins is a promising strategy for reshaping the TME and enhancing antitumor immune responses in challenging solid tumors. Future research will focus on long-term preclinical evaluation to assess the durability of the antitumor response, characterize potential late-onset toxicity and off-target effects of BifidoSumIL-2, and monitor for the development of resistance. The *Bifidobacterium* engineering toolkit is also underdeveloped as compared to other bacterial chassis. Improved availability of well-characterized genetic parts (e.g., constitutive and inducible promoters, secretion, and surface display tags), antibiotic-free maintenance strategies, and biocontainment approaches may improve strain design for human translation. In addition, although the present study used intravenous administration, the feasibility and therapeutic potential of oral delivery warrant further investigation, given its potential to enhance clinical translatability.

## MATERIALS AND METHODS

### Bacterial strains and plasmids

*B. longum* ATCC15697 was used throughout this study. The plasmid backbone (pJL21) was derived from *B. longum* MSK17.29, obtained from the symbiotic bacterial strain bank repository at the Duchossois Family Institute, University of Chicago. SumIL-2 and NanoLuc genes were inserted into the pJL21 under elongation factor Tu (P_tuf_) promoter derived from *Bifidobacterium bifidum* either with or without a secretion signal peptide derived from a hypothetical secreted protein (BLON_RS02330). Sequences for these genetic parts are included in table S1. Transformation of *B. longum* ATCC15697 was performed as previously described (*63*, *64*). Briefly, 2 ml of an overnight culture was inoculated into 100 ml of de Man, Rogosa, and Sharpe (MRS) medium containing 0.05% (w/v) cysteine HCl and 20% (w/v) glucose and incubated at 37°C until an optical density at 600 nm (OD_600_) of 0.6 to 0.9 was reached (typically 9 to 16 hours). Cultures were then chilled on ice and harvested by centrifugation at >4500 rpm for 10 min at 4°C. The cell pellet was washed twice with ice-cold wash buffer [0.5 M sucrose (Sigma-Aldrich) and 1.36 mM citric acid (Sigma-Aldrich), pH 5.8], resuspended in 2 ml of storage buffer [wash buffer supplemented with 10% glycerol (Sigma-Aldrich)] and stored at −80°C until subsequent transformation. For transformation, 50 μl of electrocompetent *B. longum* ATCC15697 cells were electroporated (25 μF, 200 Ω, 2000 V) followed by a recovery for 1 hour at 37°C in MRS medium containing 0.05% cysteine HCl. Subsequently, the transformed cells were plated onto Reinforced Clostridial Medium (RCM) agar plates containing 0.05% cysteine HCl and chloramphenicol (5 μg/ml) and cultured at 37°C. Individual colonies were streaked and validated for gene presence using colony polymerase chain reaction (PCR). The PCR products were confirmed by Sanger sequencing at the University of Chicago DNA Sequencing facility.

### Mammalian cell lines

HEK-Blue IL-2 reporter cells were purchased from InvivoGen and cultured in Dulbecco’s modified Eagle’s medium (DMEM; Gibco) supplemented with 10% heat-inactivated fetal bovine serum (FBS; Gibco), penicillin-streptomycin (Pen-Strep; 100 U/ml; Gibco), normocin (100 μg/ml; InvivoGen), and puromycin (1 μg/ml; InvivoGen). MC38 (MC38-OTI, colorectal carcinoma) and LLC cells were purchased from American Type Culture Collection, whereas B16F1 (B16F1-OVA, melanoma) was a gift from Y.-X. Fu (Tsinghua University). KPC cells, provided by the H. G. Munshi lab (Northwestern University), were isolated from spontaneous tumor tissues obtained from LSL-*Kras*^*G12D/+*^, LSL-*Trp53*^*R172H/+*^, *Pdx-1*-Cre mice (*46*). KPC-luc cells were selected as single clones with puromycin (5 μg/ml; InvivoGen, San Diego, CA) after stable infection with lentiviruses expressing luciferase. All tumor cells were cultured in DMEM supplemented with 10% heat-inactivated FBS and Pen-Strep (100 U/ml) at 37°C in 5% CO_2_ and routinely tested for mycoplasma contamination before use.

### Supernatant collection, ELISA, and in vitro bioactivity test

Wild-type *B. longum* ATCC15697 was cultured in MRS medium with 0.05% cysteine HCl and 100 mM potassium phosphate buffer (pH 7.3). The transformed *B. longum* strain was cultured in the same medium with an additional supplement of chloramphenicol (5 μg/ml). Cells were cultured in buffered MRS as medium acidification during growth in batch culture compromised the bioactivity of sumIL-2. An inoculum corresponding to an OD_600_ of ∼0.4 was cultured at 37°C for 8 hours, resulting in a final OD_600_ of around 1.0. Following culture, the samples were transferred to ice, and protease inhibitor cocktail (Sigma-Aldrich) was added at a 1:10 dilution, according to the manufacturer’s instructions. The samples were then centrifuged at 10,000 rpm for 10 min at 4°C using a precooled centrifuge. The supernatants were collected, and the centrifugation step was repeated. The resulting supernatants were stored at −20°C until further analysis.

The concentration of SumIL-2 in the supernatants was measured using a Human IL-2 ELISA kit (R&D Systems), following the manufacturer’s instructions. Before analysis, the supernatants of BifidoSumIL-2 were diluted 1:100 in Reagent buffer (R&D Systems) due to the limit of detection (15 to 1000 pg/ml). After analysis, the IL-2 concentration per OD_600_ in the supernatant was recalculated.

To assess the bioactivity of SumIL-2 secreted by BifidoSumIL-2, 50,000 HEK-Blue IL-2 reporter cells were seeded in each well of a 96-well plate containing DMEM supplemented with 10% FBS and Pen-Strep (100 U/ml). BifidoSumIL-2 supernatants, with concentrations previously determined by ELISA, were serially diluted in DMEM to obtain the desired concentrations. A 20-μl aliquot of each dilution was then added to the wells. Recombinant hIL-2 was added to separate wells at final concentrations ranging from 1 to 250 pg/ml and served as a positive control. The plate was incubated overnight at 37°C in 5% CO_2_. Next day, 20 μl of supernatant from each well was taken and added to 180 μl of prepared QUANTI-Blue Solution per well. They were further incubated at 37°C for 1 hour. The level of secreted alkaline phosphatase by HEK-Blue IL-2 reporter cells was measured by assessing the absorbance of the solution at 635 nm (OD_635_) using a spectrophotometer plate reader (Tecan Infinite 200 PRO).

### Plasmid retention assay

BifidoSumIL-2 broth cultures were diluted 1:2000 into fresh MRS medium supplemented with 0.05% cysteine HCl and 100 mM potassium phosphate buffer (pH 7.3), without chloramphenicol, and grown daily to a maximum OD_600_ of 1.9. Cultures were serially passaged each day, and 2.5 μl of 10-fold serial dilutions were plated on RCM agar with or without chloramphenicol (5 μg/ml). CFU were counted, and CFU/ml were calculated accordingly.

### In vivo tumor models

Female C57BL/6 mice were purchased from Envigo, and female *Tmem173^−/−^*, *Rag1^−/−^* and *MyD88^−/−^* mice were purchased from the Jackson Laboratory. Mice aged 6 to 10 weeks were used for all experiments. All mice experiments were conducted in accordance with the *Guide for the Care and Use of Laboratory Animals* and approved by the Institutional Animal Care and Use Committee of the University of Chicago (ACUP no. 72213). All mice were housed under specific pathogen-free conditions with a 12-hour light/12-hour dark cycle, temperatures of 20° to 23°C, and humidity of 30 to 70%.

For subcutaneous flank tumor models, MC38 and B16F1 cells (1 × 10^6^ cells in 100 μl of PBS) were subcutaneously injected into the flanks of mice. For bilateral KPC tumor model, 1 × 10^6^ KPC cells were subcutaneously injected into one flank (designated as the local tumor) and 5 × 10^5^ KPC cells into the contralateral flank (distal tumor). Tumor volumes were calculated using the formula: (length × width × height)/2 (mm^3^). Treatment was initiated when tumors reached ∼100 mm^3^. Mice were removed when individual tumor volumes exceeded 2000 mm^3^. For a metastatic LLC model, LLC cells (2 × 10^5^) were intravenously injected, and treatment was initiated on day 7.

For orthotopic PDAC model, KPC or KPC-luc cells (2 × 10^5^ to 5 × 10^5^ cells in 50 μl of PBS) were injected directly into the tail of the pancreas of mice anesthetized with isoflurane. Treatment was initiated on days 10 to 12. KPC-luc tumor growth was monitored via bioluminescence imaging using the in vivo imaging system (IVIS; Lumina XR). Mice were intraperitoneally administered d-luciferin (XenoLight) 30 min before IVIS imaging. Living Image (version 4.5, Xenogen) and Aura software were used to acquire an image sequence. The region of interest was drawn in the upper abdominal area, and the photon flux data were measured. Mice body weights were monitored throughout the experimental period as needed.

For cancer treatments, the amounts of a single dose were as follows: wild-type *B. longum*, BifidoSumIL-2, or BifidoLuci, 5 × 10^6^ CFU per mouse (equivalent to 100 μl of bacterial suspension adjusted to an OD_600_ of ∼0.1); hIL-2, 10 μg per mouse (Roche); SumIL-2–Fc, 5 μg per mouse (a gift from Y.-X. Fu); gemcitabine (Sigma-Aldrich), 100 μg per mouse; anti–PD-L1 (10F.9G2, BioXCell), anti-CD8 (2.43, BioXCell), or anti-CD4 (GK1.5, BioXCell) antibodies, 100 μg per mouse; abdominal radiation, 10 gray (XRad 225Cx). Unless otherwise specified, monotherapies were administered as follows: wild-type *B. longum* or BifidoSumIL-2 (intravenously, three times, every other day); hIL-2, SumIL-2–Fc, gemcitabine, or anti–PD-L1 (intraperitoneally, three times, every other day); and abdominal radiation (a single dose at treatment start). Combination therapies consisted of the combined doses of the respective monotherapies. A 100-μl intravenous injection of PBS served as the control and was administered on the same days as the other injections.

### Histological analysis

For LLC nodule analysis, lungs were collected 20 days post–tumor injection for histological examination. Lungs were fixed with 4% paraformaldehyde for 2 days and sent to the Human Tissue Resource Center at the University of Chicago for embedding and processing. Tissue sections were stained using a Leica Bond RX automated stainer, scanned using a CRi Pannoramic SCAN ×40 whole slide scanner, and analyzed with QuPath (v0.1.2) and ImageJ (v1.53a).

### Biodistribution of BifidoSumIL-2 and ELISA for tissue-derived IL-2

A single intravenous injection of BifidoSumIL-2 was administered to mice bearing subcutaneous B16F1 tumors that had reached ∼100 mm^3^. On 1, 3, 7, 10, and 13 days postinjection, tumors and organs (liver, spleen, kidney, heart, and lung) were collected, along with tumors and organs from untreated tumor-bearing mice as controls. Tumors and organs were placed on the ice and cut into two halves. One-half was immediately used for BifidoSumIL-2 biodistribution analysis, whereas the other was weighed and stored at −80°C for ELISA analysis.

For biodistribution analysis, samples were weighed and homogenized in 1 ml of PBS using stainless steel beads (5-mm diameter, QIAGEN) and a PowerLyzer 24 homogenizer (QIAGEN). Homogenates were serially diluted and plated on predried, prereduced RCM agar plates containing chloramphenicol (5 μg/ml), and then incubated at 37°C. Simultaneously, 100 μl of blood was collected into EDTA-treated tubes and plated on RCM agar plates containing chloramphenicol (5 μg/ml). The limit of detection is indicated in the figure.

For ELISA analysis, protease inhibitor cocktail (100×, Halt) was added according to the manufacturer’s instructions before thawing. Tumor tissues were homogenized with 1 ml of PBS. A human IL-2 ELISA kit (Invitrogen) was used for IL-2 detection. Result was calculated as a concentration of IL-2 (picograms) per tissue weight (grams).

### Biodistribution of BifidoLuci in orthotopic PDAC model

Mice bearing orthotopic KPC tumors received a single intravenous injection of BifidoLuci. For in vivo bioluminescence imaging, 100 μl of Fluorofurimazine (333 nmol, AOBIOUS) was administered intravenously before each imaging session, and body bioluminescence intensity was monitored daily from days 1 to 6 using the IVIS (Lumina XR). For biodistribution analysis, BifidoLuci was administered intravenously three times every other day to mice with orthotopic KPC tumors. Three days after the final treatment, mice received an intravenous injection of 100 μl of Fluorofurimazine, and, then, tumors and organs were collected. The collected tissues were placed in a 12-well plate and imaged using the IVIS (Lumina XR).

### Immune cell profiling

On day 7 following last treatment with PBS, hIL-2, wild-type *B. longum*, or BifidoSumIL-2, tumors and spleens were harvested from mice bearing subcutaneous B16F1 tumors or orthotopic PDAC KPC tumors and placed in isolation buffer [RPMI 1640 (VWR) supplemented with 5% heat-inactivated FBS (Gibco), Pen-Strep (100 U/ml), and 10 mM Hepes (Gibco)]. Tissues were finely cut and digested for 1 hour at 37°C with collagenase IV (1 mg/ml; Sigma-Aldrich) and deoxyribonuclease I (200 μg/ml; Sigma-Aldrich). Following digestion, cell suspensions were filtered through 70-μm cell strainers and washed in isolation buffer before staining. The following antibodies were used: CD16/CD32 (2.4G2) from BioXcell; CD45 (30-F11), CD11b (M1/70), CD4 (RM4-5), CD8α (53-6.7), NK1.1 (PK136), CD3 (17A2), CD11c (N418), F4/80 (BM8), Ly6C (HK1.4), MHCII (M5/114.15.2), PD-1 (29F.1A12), and Tim3 (RMT3-23), CD19 (ID3), CD86 (GL-1), INOS (CXNFT), and CD206 (C068C2) from BioLegend; Ly6G (1A8), NK1.1 (PK136), CD8 (53-6.7), F4/80 (T45-2342), TCF1 (S33-966), and CD11c (HL3) from BD Biosciences. Dead cells were excluded using the LIVE/DEAD Fixable Yellow Dead Cell Stain Kit (Invitrogen). Flow cytometry data were acquired using a Cytek Aurora and analyzed with FlowJo (v10.8.1).

### Liver injury test and cytokine measurement in serum and TME

Wild-type mice received a single intravenous injection of wild-type *B. longum* or BifidoSumIL-2, PBS, or intraperitoneal injections of hIL-2. Serum samples were collected at day 7 postinjections. AST/ALT assay kits from Sigma-Aldrich were used for AST/ALT detection. Results were calculated following the manufactory instructions.

For cytokine measurement, serum samples were collected from the retro-orbital venous sinus on day 7 following last treatment of PBS, hIL-2, wild-type *B. longum*, or BifidoSumIL-2. Tumors were collected on day 7 after last treatment, homogenized, and centrifuged at 12,000*g* for 10 min to collect the supernatants. All samples were supplemented with Protease inhibitor cocktails (Thermo Fisher Scientific). Inflammatory cytokines were measured using a LEGENDplex Mouse Inflammation Panel (13-plex) kit (BioLegend).

### In vitro triple coculture study, Western blot, and RT-qPCR

To generate BMDMs, bone marrow cells were flushed from the femurs and tibias of C57BL/6 wild-type or *Tmem173^−/−^* mice. Following red blood cell lysis with ammonium-chloride-potassium buffer (Corning), the remaining cells were cultured in RPMI 1640 supplemented with 10% heat-inactivated FBS, Pen-Strep (100 U/ml), recombinant mouse granulocyte-macrophage colony-stimulating factor (GM-CSF) (20 ng/ml; R&D Systems), and recombinant murine IL-4 (10 ng/ml; PeproTech). On day 4, the medium was completely replaced with fresh, prewarmed medium containing the same concentrations of GM-CSF and IL-4. On day 6, the semisuspended and loosely attached cells were harvested by gentle pipetting and used as BMDMs. For the triple coculture system, BifidoSumIL-2 (4 × 10^6^ CFU) was cocultured with BMDMs (4 × 10^5^ cells) isolated from either wild-type or Tmem173^−/−^ mice, in the presence of B16F1 cells (4 × 10^4^ cells) in vitro. After 24 hours of incubation, BMDMs were isolated using the EasySep Mouse FITC Positive Selection Kit II (STEMCELL Technologies) with a fluorescein isothiocyanate (FITC)–conjugated anti-mouse CD45 antibody (BioLegend). Total protein and RNA were then extracted for Western blot analysis and reverse transcription quantitative PCR (RT-qPCR), respectively.

Whole-cell lysates were prepared using RIPA Lysis and Extraction Buffer (Thermo Fisher Scientific) supplemented with Halt Protease Inhibitor Cocktail and Halt Phosphatase Inhibitor Single-Use Cocktail (Thermo Fisher Scientific). Equal amounts of protein were separated by 10% SDS–polyacrylamide gel electrophoresis and transferred onto polyvinylidene difluoride membranes. The membranes were blocked with 5% bovine serum albumin in Tris-buffered saline containing Tween 20 (TBST) for an hour and incubated with primary antibodies (anti-IRF3, Cell Signaling Technology; anti-pIRF3, Cell Signaling Technology; anti–α-actin, Santa Cruz Biotechnology) overnight at 4°C. After washing with TBST, the membranes were incubated with horseradish peroxidase–conjugated secondary antibodies, and signals were detected using Pierce ECL Western Blotting Substrate (Thermo Fisher Scientific).

Total RNA was extracted using the RNeasy Plus Mini Kit (QIAGEN), followed by cDNA synthesis using the High-Capacity cDNA Reverse Transcription Kit (Applied Biosystems) according to the manufacturer’s instructions. RT-qPCR was performed using SYBR Green PCR Master Mix (Applied Biosystems). Target gene expression levels were normalized to β-actin, and relative quantification was calculated using the 2^−ΔΔCT^ method. Primer sequences: mCxcl10-F, 5′-CCAAGTGCTGCCGTCATTTTC-3′; mCxcl10-R, 5′-GGCTCGCAGGGATGATTTCAA-3′; mIfnβ-F, 5′-CAGCTCCAAGAAAGGACGAAC-3′; mIfnβ-R, 5′-GGCAGTGTAACTCTTCTGCAT-3′; mIsg15-F, 5′-GGTGTCCGTGACTAACTCCAT-3′; and mIsg15-R, 5′-TGGAAAGGGTAAGACCGTCCT-3′.

### Statistical analysis

Tumor growth curves were assessed using two-way analysis of variance (ANOVA) followed by Tukey’s multiple comparisons test. Tumor burden bar graphs were analyzed using one-way ANOVA followed by Tukey’s multiple comparisons test. For other bar graphs, either one-way ANOVA or two-tailed Student’s *t* tests were applied. Data are presented as means ± SEM. Sample and replicate numbers are shown as individual data points or detailed in figure legends. GraphPad Prism was used for all statistical analyses and figure generation. A *P* value of <0.05 was considered statistically significant. Statistical significance is denoted as n.s. (not significant), *P < 0.05, **P < 0.01, ***P < 0.001, and ****P < 0.0001.
