# Supplementary material for: Engineered probiotic Bifidobacterium for tumor-targeted pancreatic cancer therapy
Source: Sci Adv. 2026 Jul 23;12(30):eadz1388. doi: 10.1126/sciadv.adz1388 (PMC13394381; doi:10.1126/sciadv.adz1388)
Supplement: Supplementary file 1 — Figs. S1 to S17 Table S1 [file sciadv.adz1388_sm.pdf]

Science Advances
AAAS

Supplementary Materials for

Engineered probiotic *Bifidobacterium* for tumor-targeted pancreatic cancer therapy

Jaehyun Lee *et al.*

Corresponding author: Ralph R. Weichselbaum, rweichselbaum@uchicagomedicine.org;
Mark Mimee, mmimee@uchicago.edu

Sci. Adv. **12**, eadz1388 (2026)
DOI: 10.1126/sciadv.adz1388

This PDF file includes:

Figs. S1 to S17
Table S1

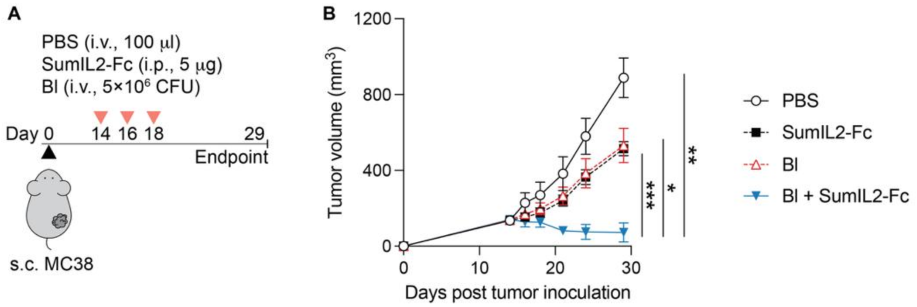

Fig. S1. *Bifidobacterium longum* and SumIL2-Fc fusion protein elicit a potent, additive antitumor effect. (A) Experimental scheme: C57BL/6 mice were injected subcutaneously (s.c.) with 1×10^6 of MC38 cells on day 0 and treated with PBS (intravenously (i.v.), 100 µl), SumIL-2-Fc fusion protein (intraperitoneally (i.p.), 5 µg) and/or *B. longum* (Bl; intravenously, 5×10^6 CFU) on days 14, 16 and 18. **(B)** MC38 tumor growth curves in mice treated with PBS, SumIL2-Fc, Bl and combination of Bl and SumIL2-Fc (n=5/group). Two-way ANOVA tests were used to analyze the tumor growth data. Data are presented as mean ± SEM. *p<0.05, **p<0.01, ***p<0.001. One of two representative experiments is shown.

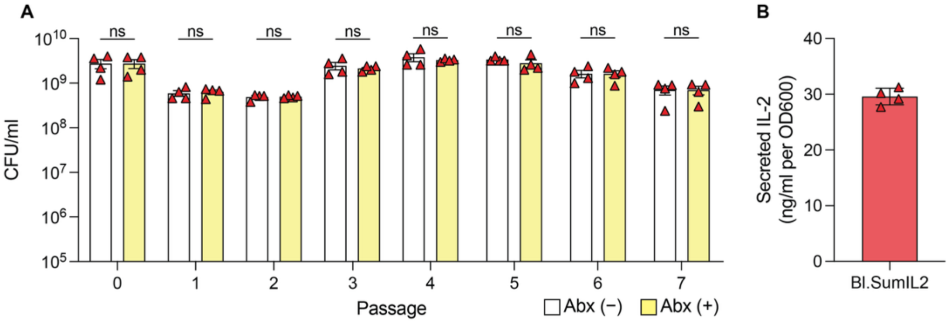

Fig. S2. Plasmid stability and secretory function of BifidoSumIL2 in the absence of antibiotic selection. (A) BifidoSumIL2 was serially passaged daily (1:2000 dilution) for seven consecutive days in culture media with or without chloramphenicol selection. To quantify plasmid loss, samples from each culture were plated on both selective and non-selective agar. Statistical analysis was performed using unpaired Student's t-tests. ns, not significant. **(B)** The concentration of SumIL2 (ng/ml) in the culture supernatant was measured by ELISA after 8 hours of growth using BifidoSumIL2 cultured under non-selective conditions for seven days. Limit of detection:15 pg/ml.

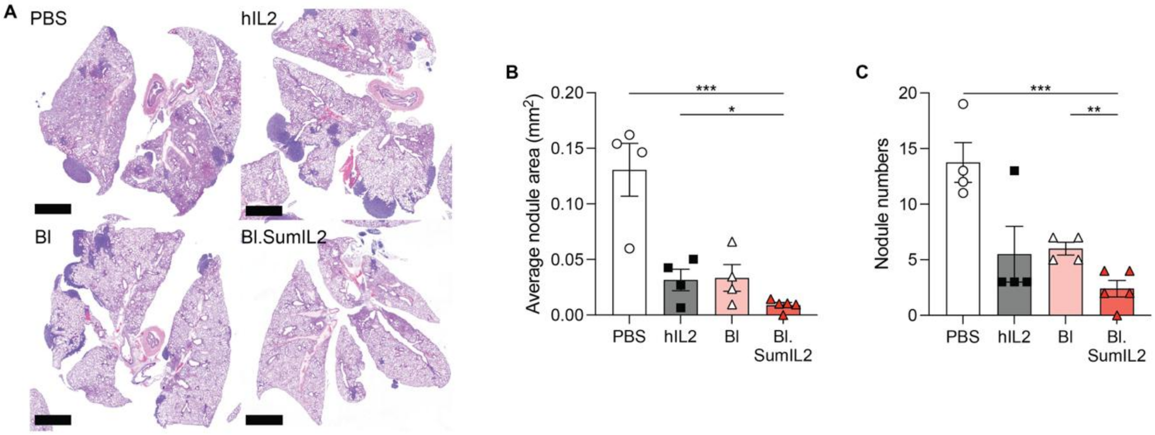

Fig. S3. BifidoSumIL2 reduces the metastatic burden in a mouse Lewis lung carcinoma model. (A) C57BL/6 mice were i.v. injected with 2×10^5 of LLC cells and treated with PBS (i.v. 100 µl), human IL-2 (i.v. 10 µg), *B. longum* (Bl; i.v. 5×10^6 CFU) or BifidoSumIL2 (Bl.SumIL2; i.v. 5×10^6 CFU) on days 7, 9 and 11. Lungs were harvested on day 20. Representative images of lungs harvested from mice treated with PBS, hIL2, Bl, or Bl.SumIL2. Scale bars represent 2 mm. **(B)** Quantification of average metastatic nodule area (mm^2) in the lungs of mice from each treatment group. **(C)** Quantification of the number of metastatic nodules in the lungs of mice from each treatment group. Data are presented as mean ± SEM. Statistical analysis was performed using Student's t-test, *p<0.05, **p<0.01, ***p<0.001. One representative of two experiments is shown.

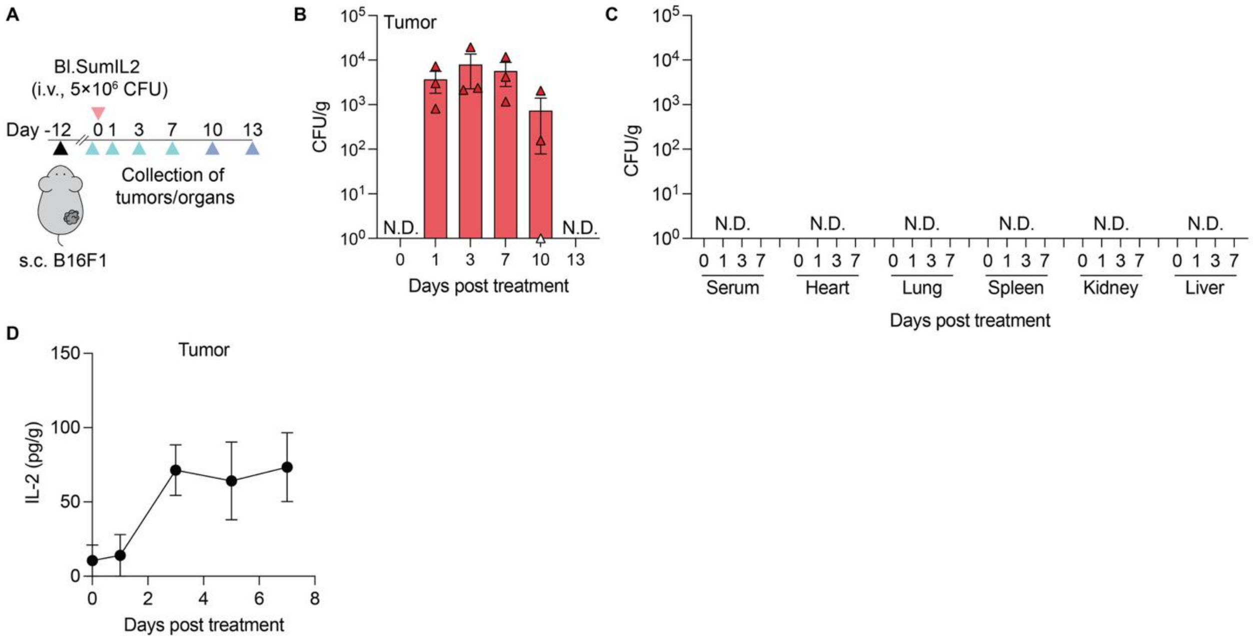

Fig. S4. *In vivo* distribution of BifidoSumIL2. (A) B16F1 tumor-bearing C57BL/6 mice were i.v. injected with 5×10^6 CFU of BifidoSumIL2 (Bl.SumIL2). Tumor, serum, heart, lung, spleen, kidney and liver samples were collected at days 1, 3, 7 post injection. To assess bacterial clearance from tumors, a separate experiment was conducted using the same protocol, and tumors were collected on days 10 and 13 post-injection. Samples from untreated tumor-bearing mice were collected as controls (Day 0). **(B, C)** Bacteria load in tumors and tissue (n=3/group). Unfilled points represent values below the limit of detection. The limit of detection was 167 CFU/ml of tissue homogenate for samples collected on days 1, 3, and 7 post-injection, and 33.3 CFU/ml for samples collected on days 10 and 13 post-injection. **(D)** IL2 level in tumors (n=4). Data are presented as mean ± SEM. One of two representative experiments is shown. N.D., not detected.

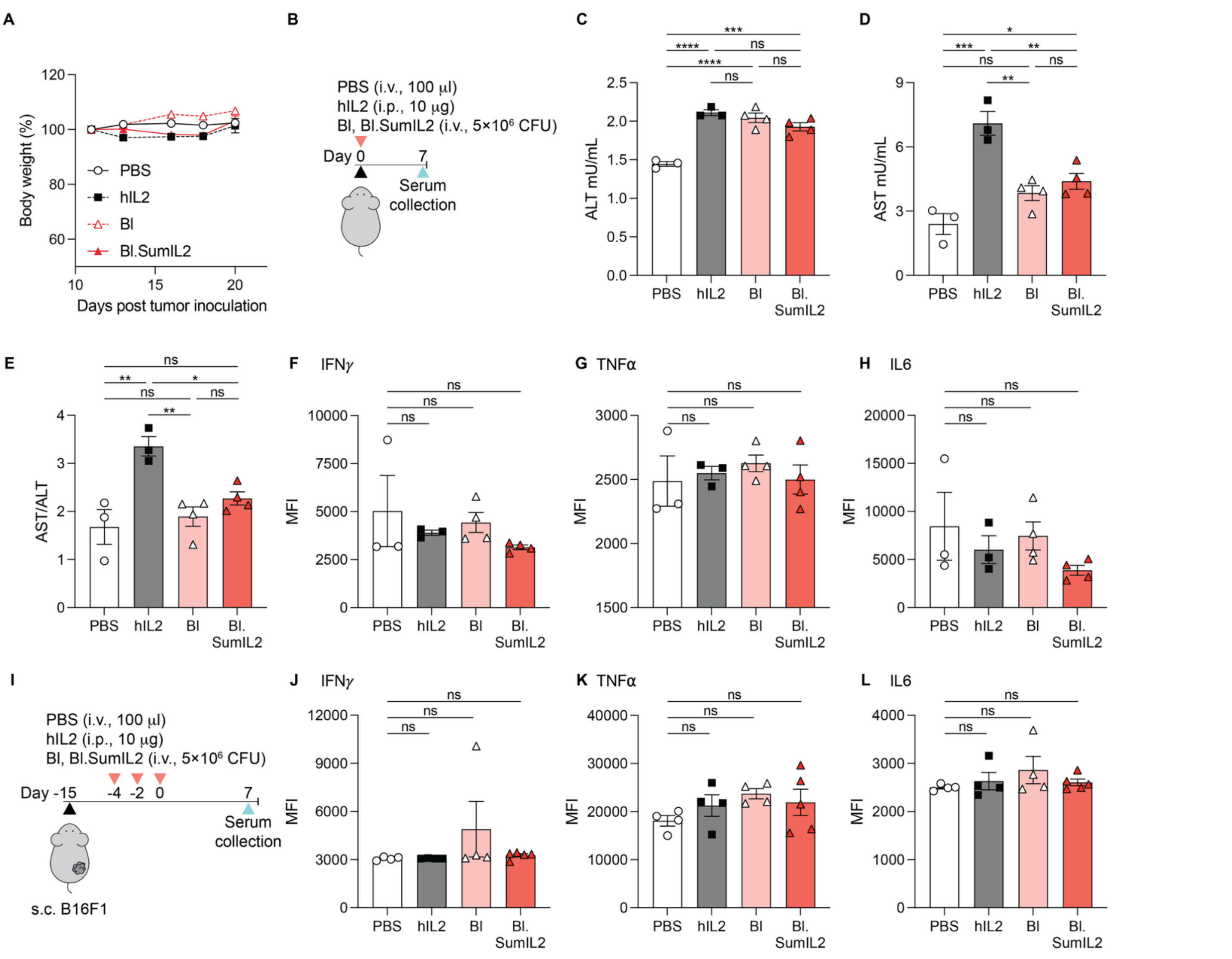

Fig. S5. Safety evaluation of BifidoSumIL2 in mice. (A) C57BL/6 mice were s.c. injected with 1×10^6 B16F1 cells on day 0 and treated with PBS (i.v., 100 µl), hIL2 (i.p., 10 µg), wild-type B. longum (Bl; i.v., 5×10^6 CFU) or BifidoSumIL2 (Bl.SumIL2; i.v., 5 × 10^6 CFU) on days 11, 13 and 15. Body weight changes are shown over 9 days after the first treatment (n=3 per group). **(B–H)** C57BL/6 mice were treated once with PBS (i.v., 100 µl), human IL-2 (i.v., 10 µg), B. longum (i.v., 5×10^6 CFU) or BifidoSumIL2 (i.v., 5×10^6 CFU). Serum samples were collected 7 days after treatment. Levels of aspartate transaminase (AST) **(C)**, alanine transaminase (ALT) **(D)**, and the AST/ALT ratio **(E)** were shown as indicators of liver injury. Serum IFNγ **(F)**, TNFα **(G)** and IL6 **(H)** levels (mean fluorescence intensity, MFI) **(I–L)** Tumor-bearing C57BL/6 mice (B16F1; 1 × 10⁶ cells, s.c., day -15) were treated with PBS (i.v., 100 µl), hIL2 (i.p., 10 µg), wild-type B. longum (Bl; i.v., 5 × 10⁶ CFU) or BifidoSumIL2 (Bl.SumIL2; i.v., 5×10⁶ CFU) on days -4, -2 and 0. Serum samples were collected 7 days after the last treatment. Serum IFNγ **(J)**, TNFα **(K)** and IL6 **(L)** level (MFI). **(C-H, J-L)** Statistical significance was determined by one-way ANOVA. Data are presented as mean ± SEM. ns, not significant; *$p < 0.05$, **$p < 0.01$, ***$p < 0.001$, ****$p < 0.0001$.

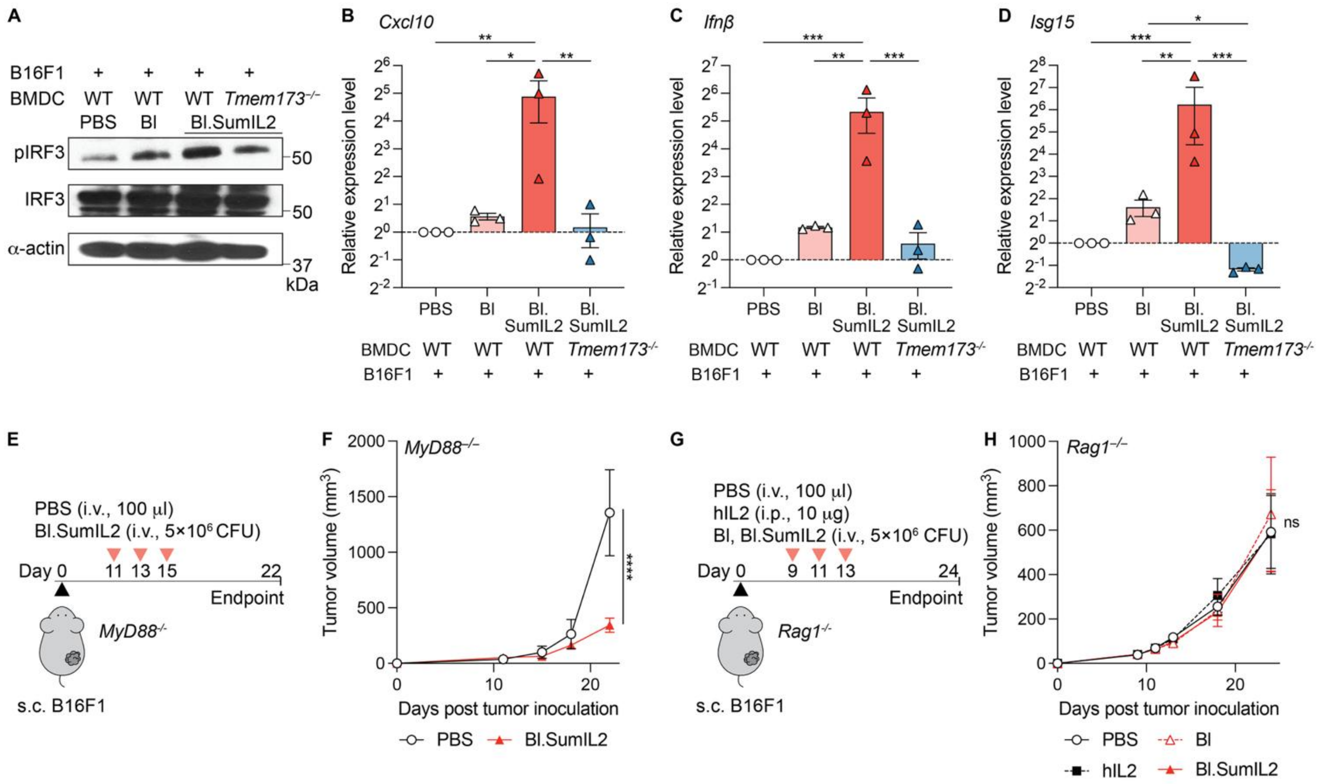

Fig. S6. BifidoSumIL2 activates the innate STING pathway and its antitumor effect depends on the adaptive immune system but is independent of MyD88. (A-D) 4×10⁶ CFU of BifidoSumIL2 were co-cultured with 4×10⁵ cells bone marrow-derived myeloid cells (BMDMs) from either wild-type or Tmem173$^{-/-}$ mice in the presence of 4×10⁴ cells B16F1 tumor cells in vitro. After 24 hours, BMDMs were isolated and then total protein and RNA were extracted for western blot analysis **(A)** or qPCR of *Ifnb* **(B)**, *Cxcl10* **(C)**, and *Isg15* **(D)** genes. **(B-D)** Gene expression levels were normalized to the PBS control. One-way ANOVA was used for statistical analysis. **(E, F)** *Myd88*$^{-/-}$ mice were s.c. injected with 1×10⁶ of B16F1 cells on day 0 and i.v. treated with PBS (100 µl) or BifidoSumIL2 (Bl.SumIL2; 5×10⁶ CFU) on days 11, 13 and 15 (n=4/group). **(G, H)** *Rag1*$^{-/-}$ mice were s.c. injected with 1×10⁶ of B16F1 cells and treated with PBS, hIL2, wild type *B. longum* or BifidoSumIL2 on Days 9, 11 and 13 (n=4-5/group). **(F, H)** Mean tumor growth curves for the tumor-bearing mice. Two-way ANOVA tests were used to analyze the tumor growth data. Data are presented as mean ± SEM. ns, not significant, *p<0.05, ****p<0.0001.

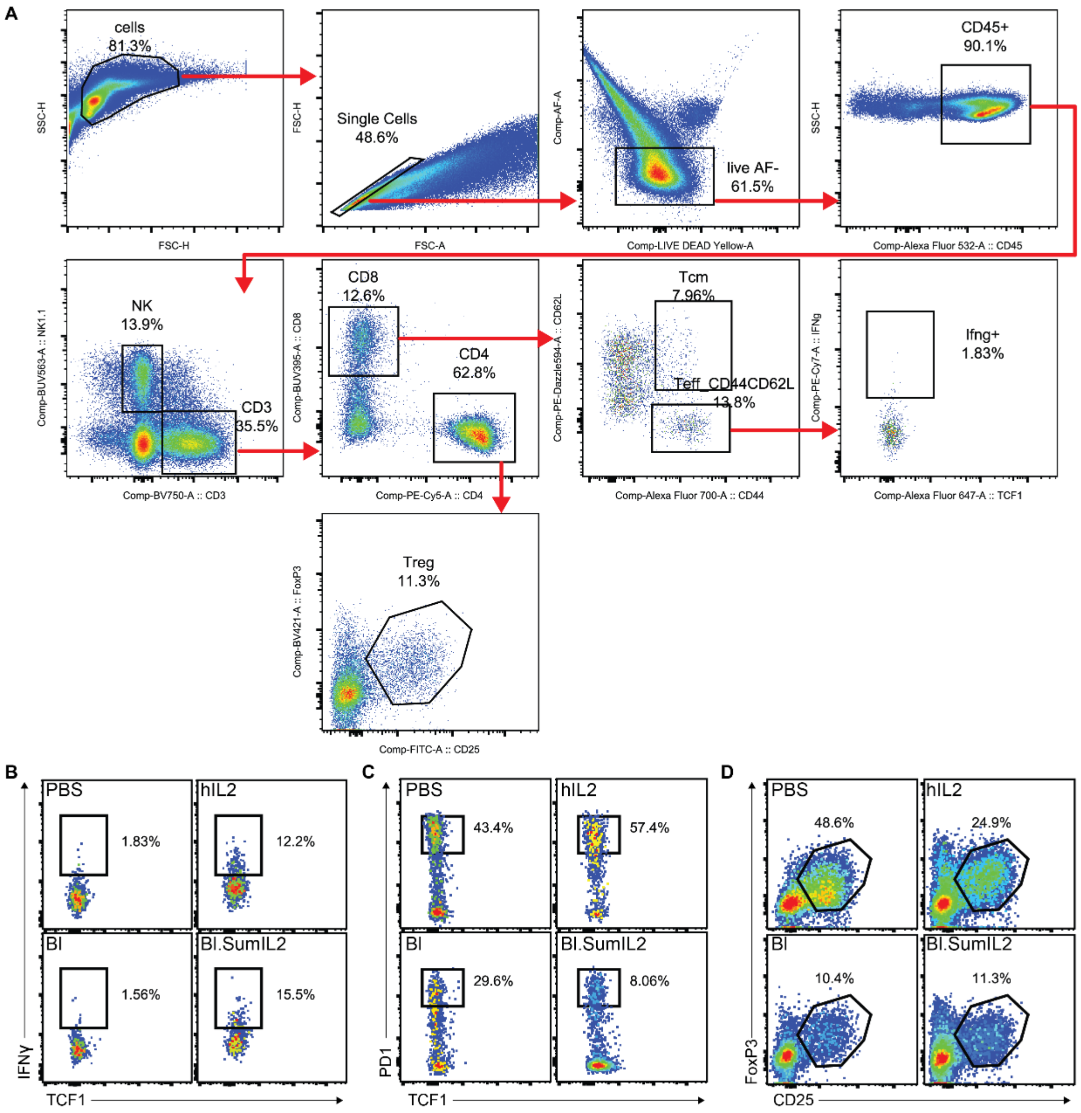

Fig. S7. Identification of T cell populations in the TME post-BifidoSumIL2 treatment. (A) Gating strategy for the immune cell populations. FSC-H, forward scatter height; FSC-A, forward scatter area; SSC-H, side scatter height; AF, autofluorescence. **(B-D)** Representative dot plots for gating of Teff, Treg and Tex cells. **(B)** Percentages of IFNg^{+}Teff cells, IFNg^{+} T cells gated on CD45^{+}CD3^{+}CD8^{+}CD44^{+}CD62L^{-} cells in the TME post treatment. **(C)** Percentages of Tex cells, PD-1^{hi} T cells gated on CD45^{+}CD3^{+}CD8^{+} cell in the TME post treatment. **(D)** Percentages of Treg cells, FoxP3^{+}CD25^{+} T cells gated on CD45^{+}CD3^{+}CD4^{+} cells in the TME post treatment.

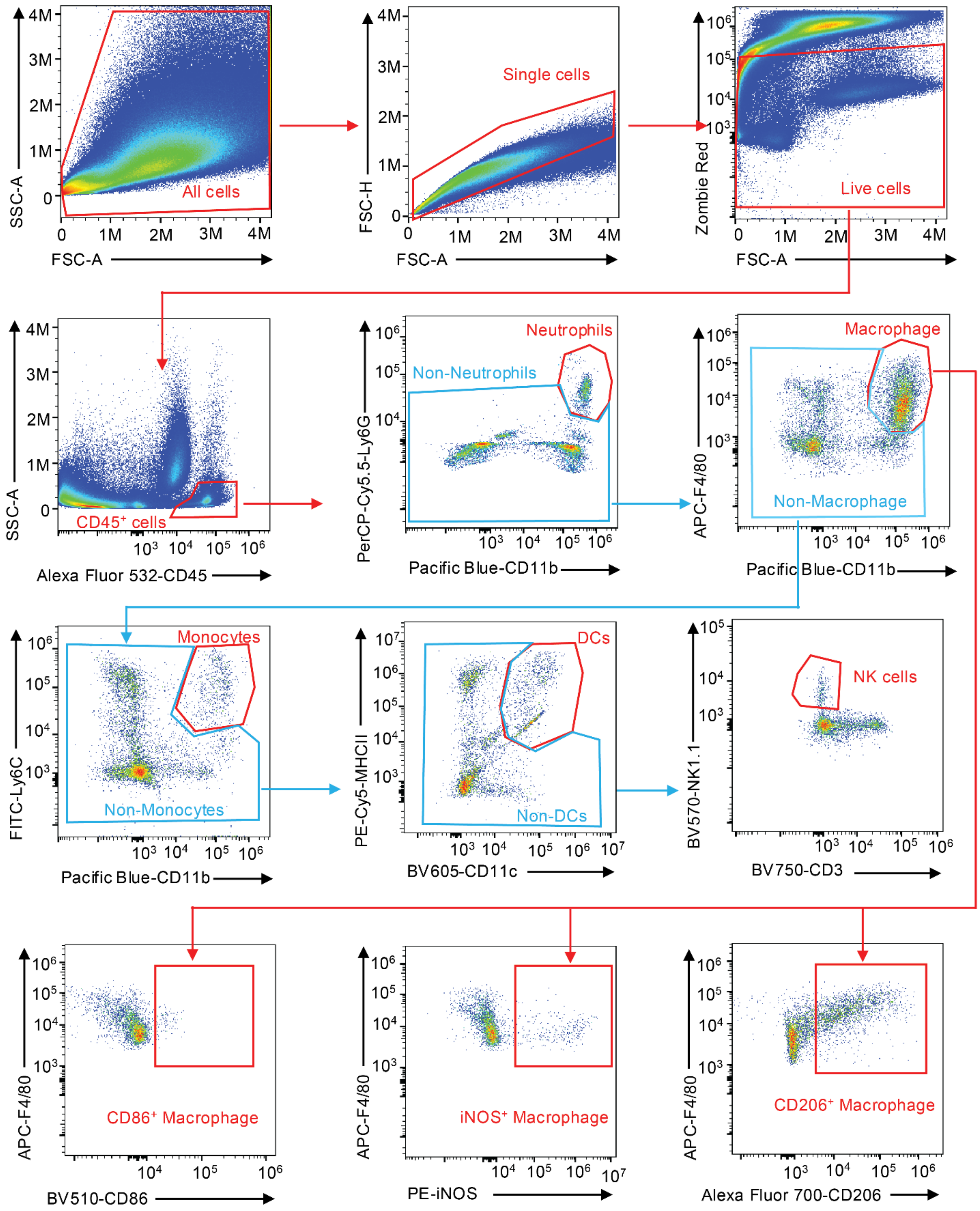

Fig. S8. Identification of immune cell populations in the TME post-BifidoSumIL2 treatment. Gating strategy for the immune cell populations. FSC-H, forward scatter height; FSC-A, forward scatter area; SSC-H, side scatter height.

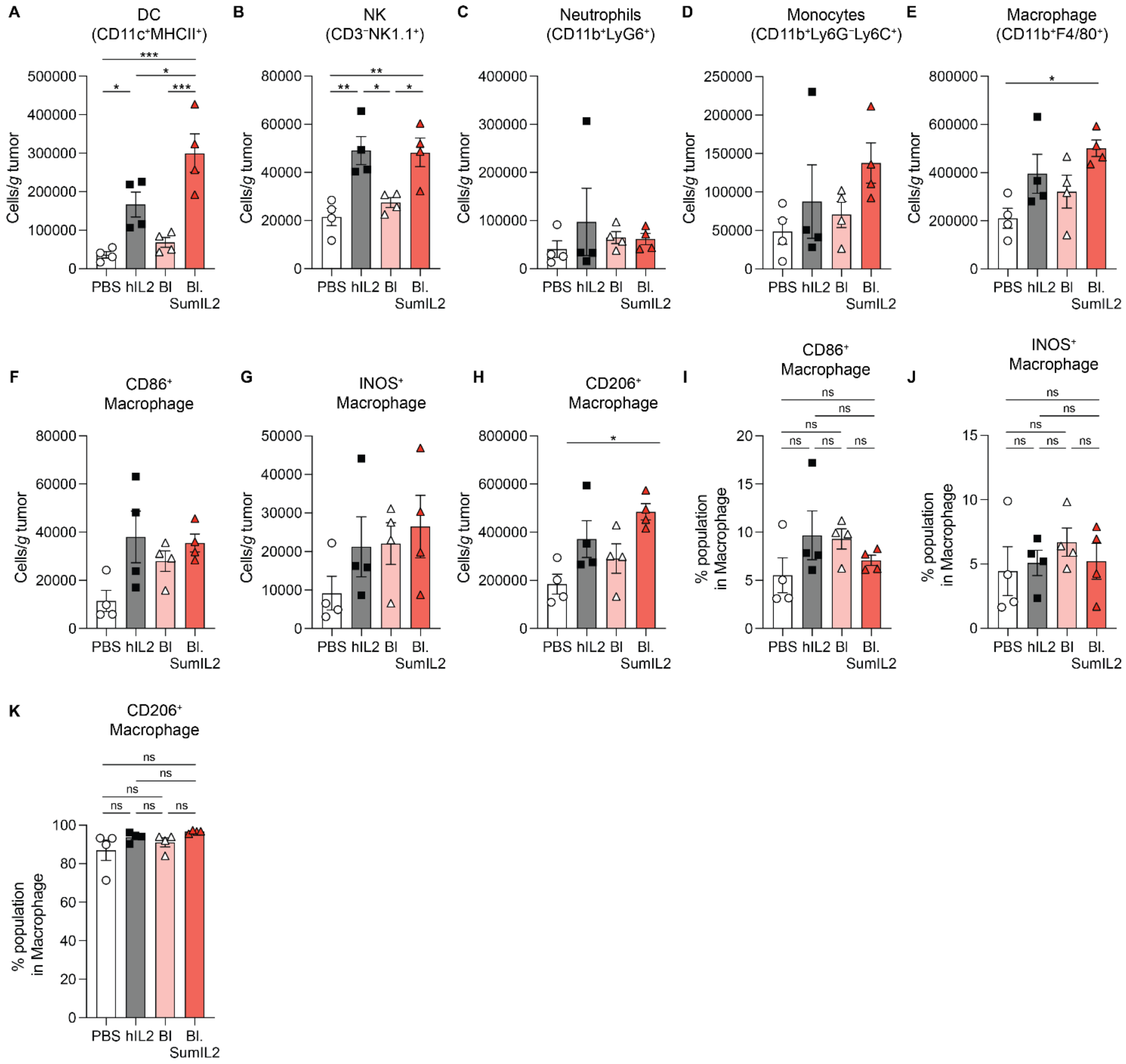

Fig. S9. BifidoSumIL2 modulates immune cell populations in the TME. C57BL/6 mice were s.c. injected with 1×10^{6} of B16F1 cells on day 0 and treated with PBS (i.v., 100 µl), hIL2 (i.p., 10 µg), wild type *B. longum* (Bl; i.v., 5×10^{6} CFU) or BifidoSumIL2 (Bl.SumIL2; i.v., 5×10^{6} CFU) on days 11, 13 and 15. Tumors were harvested 7 days after the final treatment (n=4/group). The number of individual immune cell populations (cells/g tumor). Statistical significance was determined by one-way ANOVA. Data are presented as mean ± SEM. **(A–H)** Comparisons not indicated were not significant. *p < 0.05, **p < 0.01, ***p < 0.001, ****p < 0.0001. **(J–L)** ns, not significant.

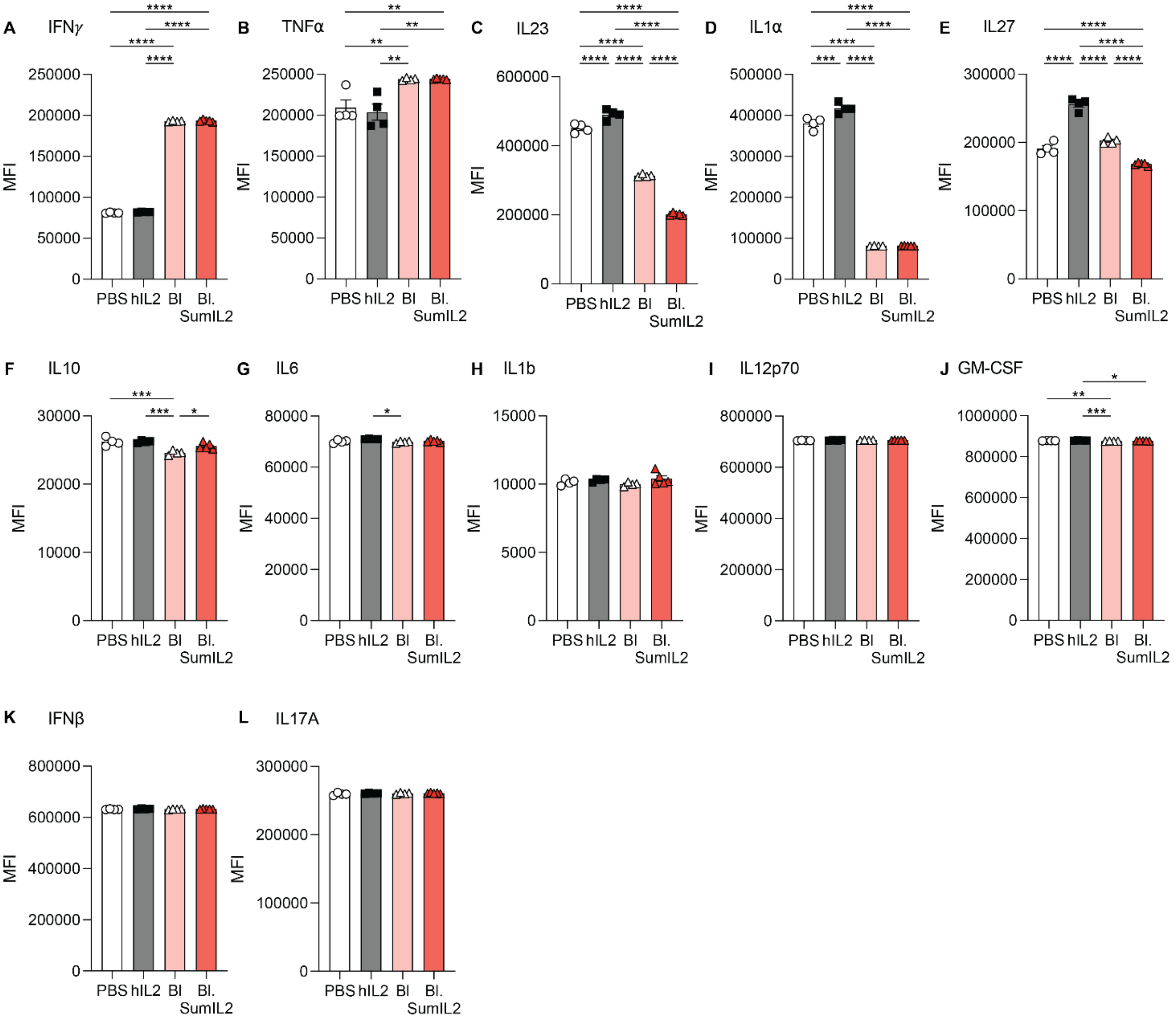

Fig. S10. BifidoSumIL2 modulates cytokine profiles in the TME. (A-M) C57BL/6 mice were s.c. injected with 1×10^6 of B16F1 cells on day 0 and treated with PBS (i.v., 100 µl), hIL2 (i.p., 10 µg), wild type *B. longum* (Bl; i.v., 5×10^6 CFU) or BifidoSumIL2 (Bl.SumIL2; i.v., 5×10^6 CFU) on days 11, 13 and 15. Tumors were harvested 7 days after the final treatment (n=3-4/group). Serum cytokine levels (mean fluorescence intensity, MFI). Statistical significance was determined by one-way ANOVA. Data are presented as mean ± SEM. Comparisons not indicated were not significant. *p < 0.05, **p < 0.01, ***p < 0.001, ****p < 0.0001.

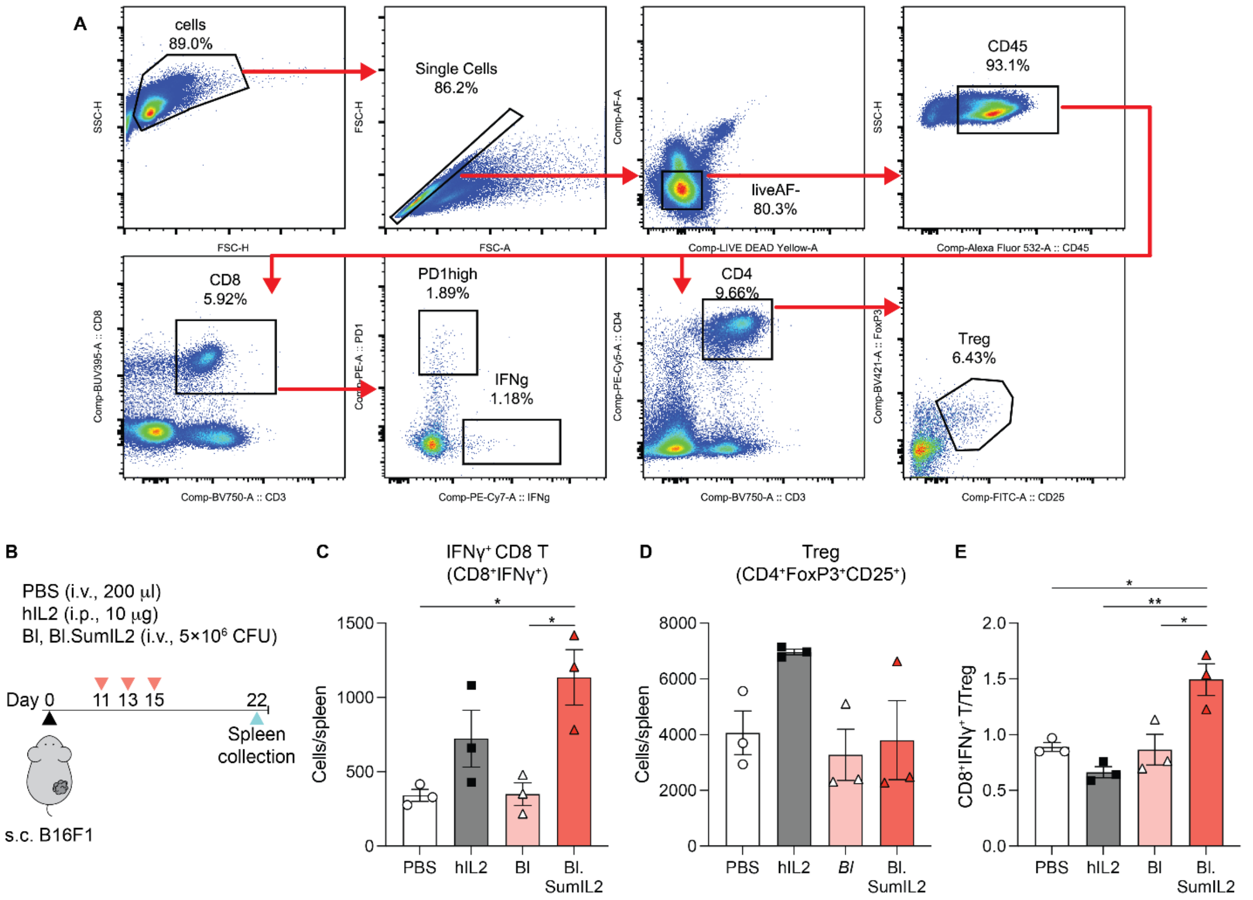

Fig. S11. BifidoSumIL2 modulates the splenic T cell population. (A) Gating strategy for the identification of immune cell populations in the mouse spleen. **(B-E)** C57BL/6 mice were s.c. injected with 1×10^6 of B16F1 cells on day 0 and treated with PBS (**i**.v.**,** 100 µl), hIL2 (i.p., 10 µg), wild type *B. longum* (Bl; i.v., 5×10^6) or BifidoSumIL2 (Bl.SumIL2; i.v., 5×10^6 CFU) on on days 11, 13 and 15. Spleens were harvested 7 days after the final treatment (n=3/group). **(C–E)** T cell subsets were gated on $CD45^+CD3^+$ cells. The number of $IFN\gamma^+CD8^+$ T cells **(C)** and Tregs ($FoxP3^+CD25^+CD4^+$) **(D)**, and the $IFN\gamma^+CD8^+$ T/Treg ratio **(E)** are shown. **(C-E)** One-way ANOVA tests were used to compare groups. Data are presented as mean ± SEM. *p<0.05, **p<0.01. One of two representative experiments is shown.

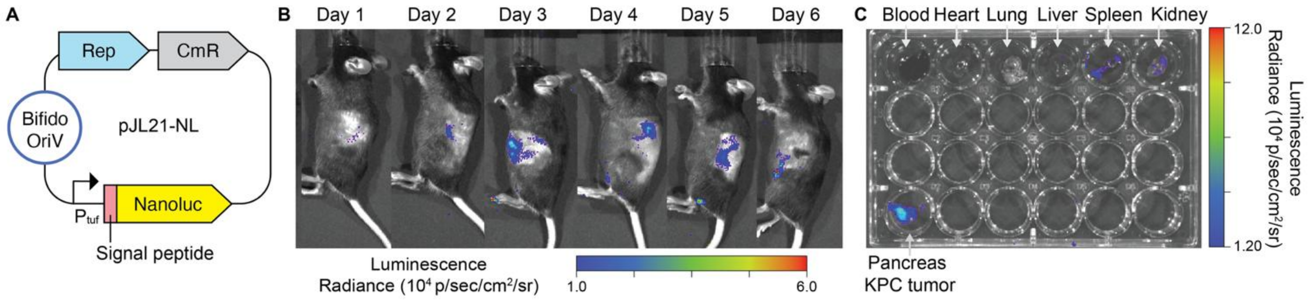

Fig. S12. BifidoLuci colonize and can release a payload within orthotopic PDAC tumors. (A) Plasmid construction for NanoLuc secretion. A *Bifidobacterium* constitutive promoter (P_{tuf}) was placed upstream of the NanoLuc gene, along with a secretion signal peptide derived from a hypothetical protein (BLON_RS02330) into the pJL21 plasmid. **(B)** Representative bioluminescence images showing the intensity in orthotopic KPC tumor-bearing mice following a single intravenous administration of BifidoLuci (5×10^6 CFU), monitored daily for 6 days. One experiment is shown. **(C)** Representative bioluminescence images of organs and KPC tumors harvested 3 days after the third intravenous administration of BifidoLuci in orthotopic KPC tumor-bearing mice. One of two experiments is shown.

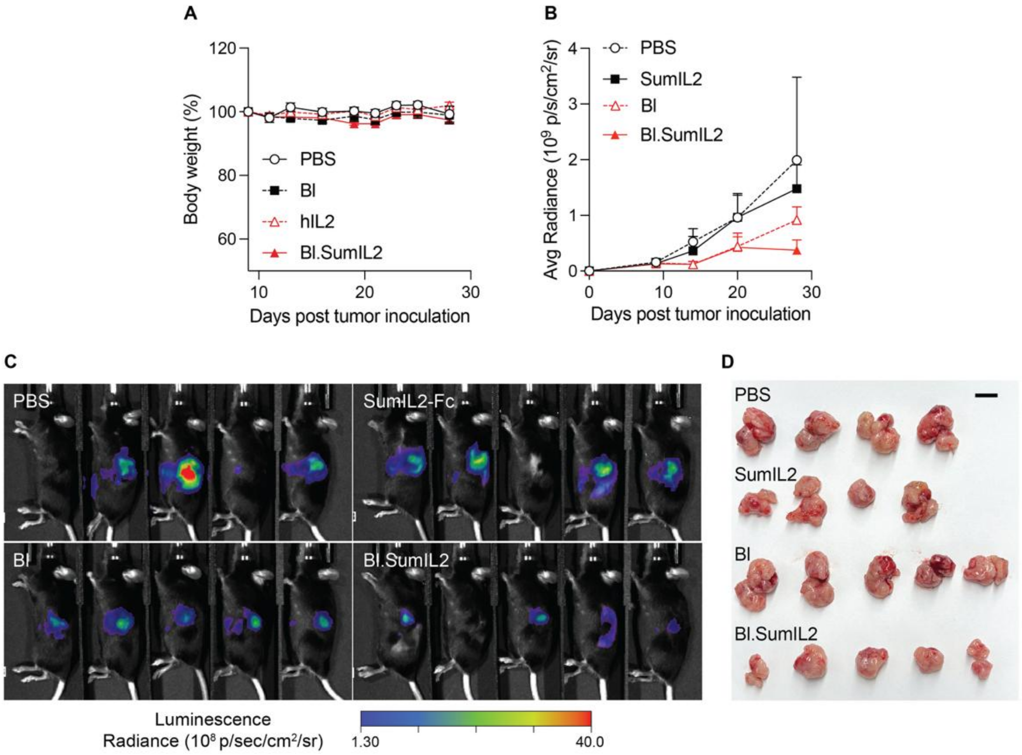

Fig. S13. BifidoSumIL2 restrains orthotopic KPC tumor growth. C57BL/6 mice were injected with 5×10^5 cells of KPC-luc cells in the pancreas tail on Day 0 and treated with PBS (i.v., 100 µl), SumIL2-Fc (i.p., 5 µg), wild-type *B. longum* (Bl; i.v., 5×10^6 CFU) or BifidoSumIL2 (Bl.SumIL2; i.v., 5×10^6 CFU) on days 9, 11 and 13 (n=5/group). **(A)** Body weight changes over time in mice. **(B)** Bioluminescence signal over time in mice treated with PBS, SumIL2-Fc, wild-type *B. longum* or BifidoSumIL2. **(C)** Representative bioluminescence images taken on day 28 after tumor inoculation. **(D)** Representative tumor images at the end of the study (day 33). Scale bar, 1 cm.

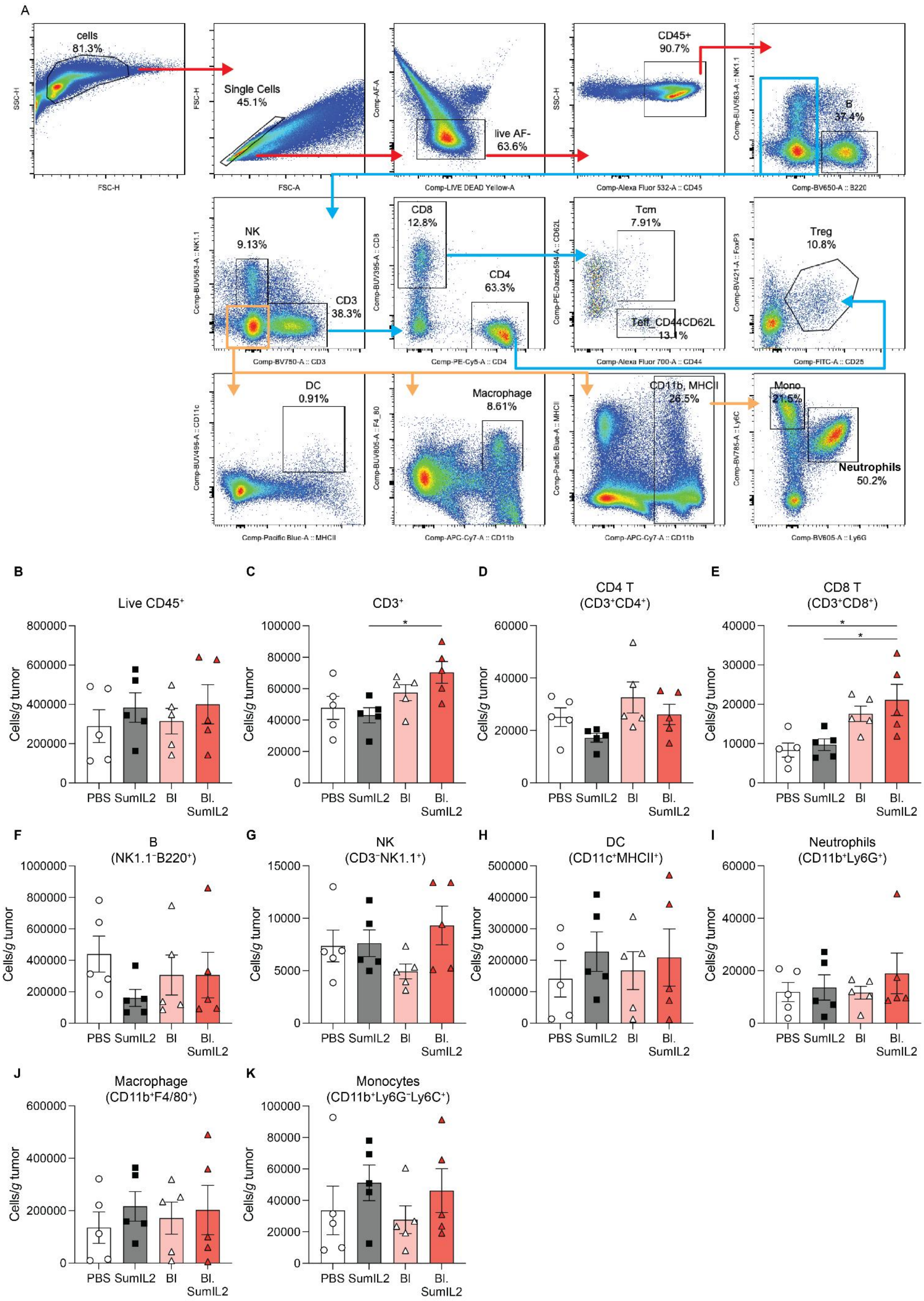

A
cells
81.3%
Single Cells
45.1%
live AF-
63.6%
CD45+
90.7%
B
37.4%
NK
9.13%
CD3
38.3%
CD8
12.8%
CD4
63.3%
Tcm
7.91%
Teff_CD44CD62L
13.1%
Treg
10.8%
DC
0.91%
Macrophage
8.61%
CD11b, MHCII
26.5%
Mono
21.5%
Neutrophils
50.2%
SSC-H
FSC-H
FSC-A
Comp-AF-A
Comp-LIVE DEAD Yellow-A
Comp-Alexa Fluor 532-A :: CD45
Comp-BUV563-A :: NK1.1
Comp-BV650-A :: B220
Comp-BV750-A :: CD3
Comp-BUV395-A :: CD8
Comp-PE-Cy5-A :: CD4
Comp-PE-Dazzle594-A :: CD62L
Comp-Alexa Fluor 700-A :: CD44
Comp-BV421-A :: FoxP3
Comp-FITC-A :: CD25
Comp-BUV496-A :: CD11c
Comp-Pacific Blue-A :: MHCII
Comp-BUV805-A :: F4_80
Comp-APC-Cy7-A :: CD11b
Comp-Pacific Blue-A :: MHCII
Comp-BV785-A :: Ly6C
Comp-BV605-A :: Ly6G
B
Live CD45+
C
CD3+
D
CD4 T
(CD3+CD4+)
E
CD8 T
(CD3+CD8+)
F
B
(NK1.1−B220+)
G
NK
(CD3−NK1.1+)
H
DC
(CD11c+MHCII+)
I
Neutrophils
(CD11b+Ly6G+)
J
Macrophage
(CD11b+F4/80+)
K
Monocytes
(CD11b+Ly6G−Ly6C+)
Cells/g tumor
PBS
SumIL2
Bl
Bl.
SumIL2
*

Fig. S14. Spectral flow cytometry analysis of tumor-infiltrating immune cells in orthotopic KPC tumors. C57BL/6 mice were injected with 5×10^5 cells of KPC-luc cells in the pancreas tail on Day 0 and treated with PBS (i.v., 100 µl), SumIL2-Fc (i.p., 5 µg), wild-type *B. longum* (Bl; i.v., 5×10^6 CFU) or BifidoSumIL2 (Bl.SumIL2; i.v., 5×10^6 CFU) on days 9, 11 and 13 (n=5/group). Tumors were harvested 7 days after the final treatment **(A)** Gating strategy for the immune cell populations. FSC-H, forward scatter height; FSC-A, forward scatter area; SSC-H, side scatter height; AF, autofluorescence. **(B-K)** The number of individual immune cell populations (cells/g tumor). Statistical significance was determined by one-way ANOVA. Data are presented as mean ± SEM. (B–I) Comparisons not indicated were not significant. *p < 0.05, **p < 0.01, ***p < 0.001, ****p < 0.0001. **(J–L)** ns, not significant.

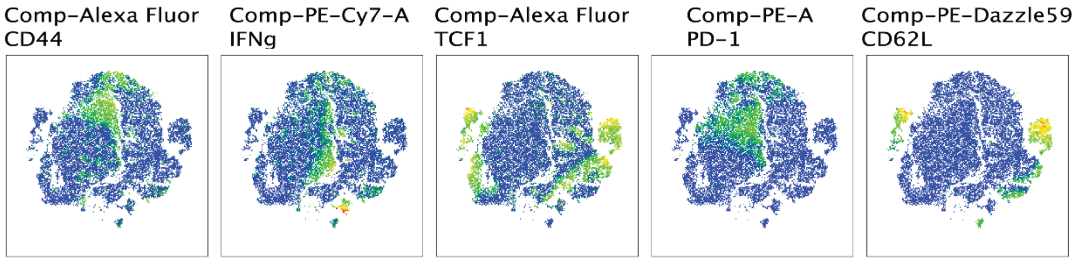

Fig. S15. Spectral flow cytometry analysis of tumor-infiltrating CD8$^+$ cells s in orthotopic KPC tumors. Spectral flow cytometry and unsupervised t-SNE clustering of 48,000 live CD8$^+$ cells from orthotopic KPC tumor samples of mice treated with PBS, Sum IL-2, *B. longum* or BifidoSumIL2. 6 distinct clusters or populations of immune cells were identified, according to expression of cell type-specific markers.

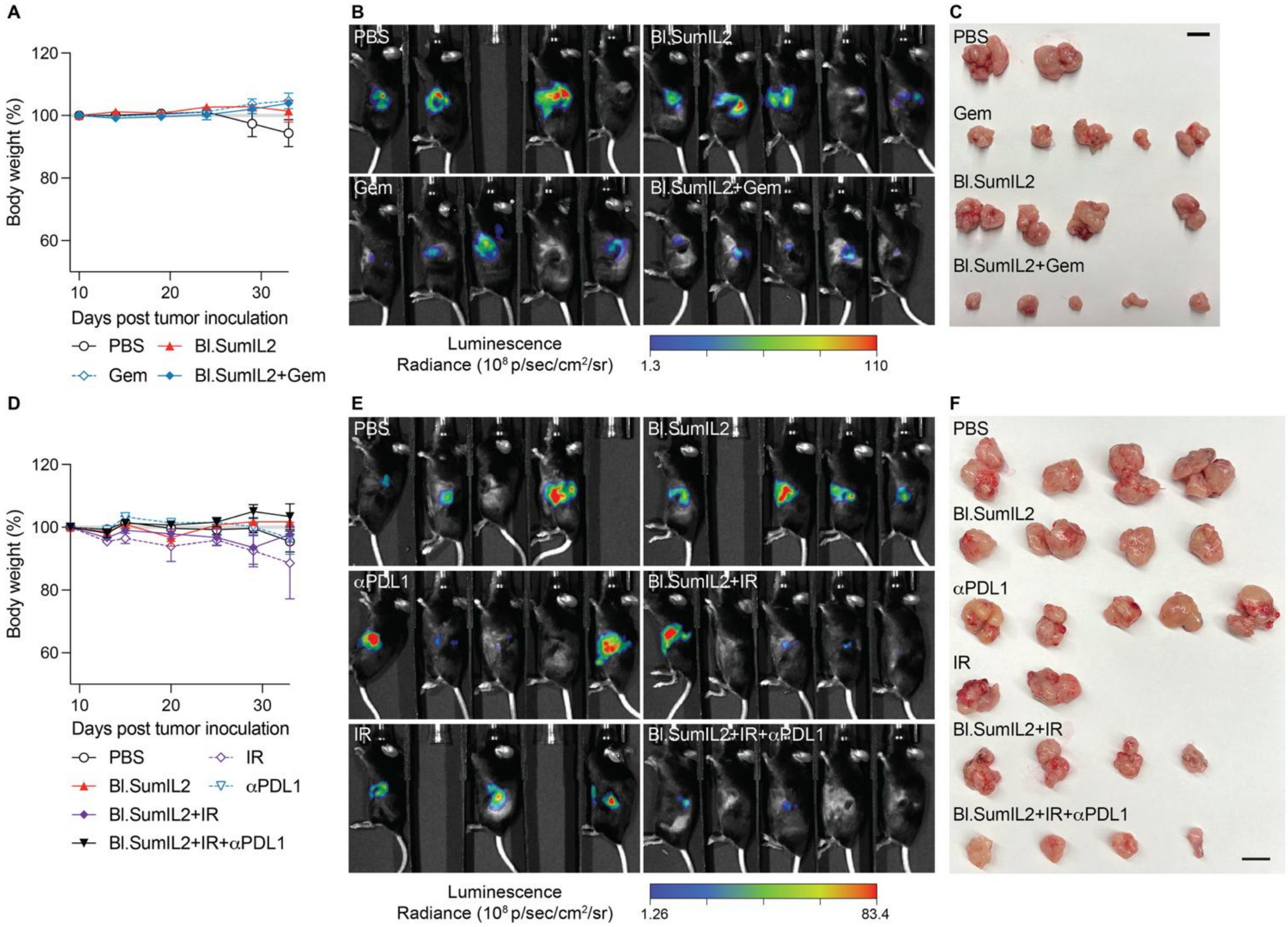

Fig. S16. Body weight changes and representative results of BifidoSumIL2 in combination with chemotherapy, immunotherapy, and abdominal irradiation in an orthotopic PDAC model. **(A-C)** Mice were injected with KPC-luc cells in the pancreas tail on Day 0 and were treated with PBS (**i**.v.**,** 100 µl), BifidoSumIL2 (Bl.SumIL2; i.v., 5×10^6 CFU) and/or gemcitabine (Gem; i.p., 100 µg) on days 10, 12 and 14 (n=5/group). **(A)** Body weight changes over time in mice. **(B)** Representative bioluminescence images taken on day 33 after tumor inoculation **(C)** Representative tumor images at the end of the study (day 35). Scale bar, 1 cm. **(D-F)** Mice were injected with KPC-luc cells in the pancreas tail on Day 0 and were treated with PBS (**i**.v.**,** 200 µl), BifidoSumIL2 (Bl.SumIL2; i.v., 5×10^6 CFU) and/or αPDL1 (i.p., 100 µg) on days 11, 13 and 15, and/or abdominal irradiation (IR; 10 Gy) on days 11 (n=5/group). **(D)** Body weight changes over time **(E)** Representative bioluminescence images taken on day 29 after tumor inoculation **(F)** Representative tumor images at the end of the study (day 33). Scale bar, 1 cm. One of two representative experiments is shown.

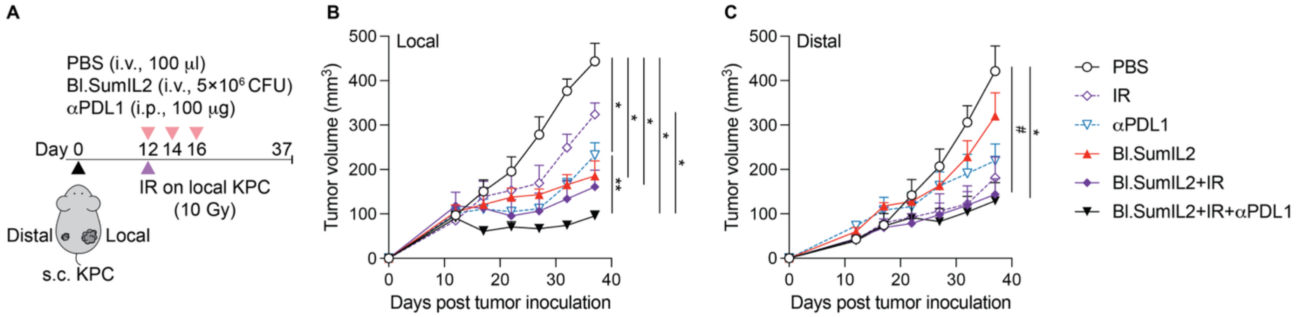

Fig. S17. Triple combination enhances antitumor efficacy in a bilateral KPC tumor model. (A) C57BL/6 mice were subcutaneously (s.c.) injected with 1×10^6 KPC cells into one flank (designated as the local tumor) and 5×10^5 KPC cells into the contralateral flank (distal tumor) on Day 0. Mice were treated with PBS (i.v., 100 µl), BifidoSumIL2 (Bl.SumIL2; i.v., 5×10^6 CFU), anti–PD-L1 antibody (i.p., 100 µg), and/or irradiation (IR; 10 Gy applied to the local tumor only) on Days 12, 14, and 16 (n=5 per group). Tumor growth curves of locally irradiated tumors **(B)** and distal, non-irradiated tumors **(C)**. **(B, C)** Two-way ANOVA tests were used to analyze the tumor growth. Data are presented as mean ± SEM. *p<0.05, **p<0.01, #P=0.532.

Name	Sequence
Plasmid backbone (pJL21)	ACGTGATGGACAACTACAGGAGCCTCGAGGACCTCAAGACCACCGTCACCAAGGCCCTGG<br>AACACAACGTCAGAACCAGCGATGGCATGAACTGGAACCTGCACGACCTGGTGTACGAGG<br>CGCTGAGCGAGGAATGGGGCAGAAGGGACGGCGAGATCAGCGACCTCTGGGCGGACGAC<br>GGGCCAAGCGGATACCAGCCACCCTCATACGAGCCGGTCAACCCCGAACGCAGGACTCCC<br>CAAACACCCTCCGATGGCCTGATCTGACGTCCAAAAAAAGGCGCCGTGCGCCCTTTTTAAA<br>TCTTTTAAAATCTTTTTACATTCTTTTAGGCCCTCCGCAGCCCTTGGAAACATTGGGCTCAGA<br>GGATGTTACTGGGGACAAAAAGGGAGCGAACCGGGGACAAAAAGGGAGCGAACCGGGGA<br>CAAAAAGGGAGCGAACCGGGGACAAAAAGGGAGCGAACCGGGGACGTTGCTAAAATGTG<br>TCTCCTTTTTGATCAAGGTGGGGACTCAAATTATTTGTGGACTAACTTAATTTGAGTCCCCCA<br>TAGGAGCTATGCTAAGGCCATGTCCAATGAGATCGTGAAGTTCAGCAACCAGTTCAACAAC<br>GTGGCACTGAAGAAGTTCGACGCCGTGCACCTGGACGTGCTCATGGCGATCGCCTCAAGG<br>GTGAGGGAGAAGGGCACGGCCACGGTGGAGTTCTCGTTCGAGGAGCTGCGCGGCCTCATG<br>CGGCTGAGGAAGAACCTGACCAACAGGCAGCTGGCCGACAAGATCGTGCAGACGAACGC<br>GCGGCTGCTGGCGTTGAACTACATGTTCGAGGATTCGGGCAAGATCATCCAGTTCGCGCTG<br>TTCACGAAGTTCGTTACCGACCCGCAGGAGGCGACCCTCGCGGTTGGGGTCAACGAGGAG<br>TTCGCGTTCCTGCTCAACGACCTGACCAGCCAGTTCACGCGCTTCGAGCTGGCCGAGTTCG<br>CCGACCTCAAAAGCAAGTACGCCAAGGAGTTCTACCGCAGGGCCAAACAATACCGCAGCT<br>CCGGAATCTGGAAGATCAGCCGCGACGAGTTCTGCCGACTGCTCAGCGTTCCCAAATCCAC<br>AGCCGAGCAAGTGAGAGATCTCAACAAACGAGTCCTCAAGCCGATTATCGAGGAGTGTGG<br>GCCACTCCTTGGACTGAAGATCGAGCGCCAGTACGTGAAACGCAGGCTGTCGGGGTTCGT<br>GTTCACGTTCGCCCGCGAGACCCCTCCGGTGATCGACGCCAGGCCCGTGGAGGCGAGGAA<br>GGCGGAGGATGCGGGCCATTGGACGAGCGTCGCCGGGTACGGCGAGGTGTTCACGACCAC<br>TGAGCTGTTCGACGTGACGGCCGCGCGTGACCACTTCGACGGCACCGTGGAGGCCGGGGA<br>ATGCCGTTTCTGCGCGTTTGACGCTCGCAACCGCGAACATCATGCGCAGAACGCCGGAAAG<br>CTGTTCTAGCGGCCGTGTCCGCGCCTCTGGGGCGGTTGCGCGCTCCATGGGTTGAT
Antibiotic selection (*cat* gene)	TACCGCTGACTGGGTTCTGGCTCCAAAGTGGCAAGAAACCCAGAAACCCAGTACAATGTA<br>GCGAGTGTCTTGTGCCGCAAGCGCAAGCCAACTACACCCCTGACCCAAGGAGAACATCAT<br>GGAGAAAAAAATCACTGGATATACCACCGTTGATATATCCCAATGGCATCGTAAAGAACATT<br>TTGAGGCATTTCAGTCAGTTGCTCAATGTACCTATAACCAGACCGTTCAGCTGGATATTACG<br>GCCTTTTTAAAGACCGTAAAGAAAAATAAGCACAAGTTTTATCCGGCCTTTATTCACATTCT<br>TGCCCGCCTGATGAATGCTCATCCGGAATTTCGTATGGCAATGAAAGACGGTGAGCTGGTG<br>ATATGGGATAGTGTTCACCCTTGTTACACCGTTTTCCATGAGCAAACTGAAACGTTTTCATC<br>GCTCTGGAGTGAATACCACGACGATTTCCGGCAGTTTCTACACATATATTCGCAAGATGTGG<br>CGTGTTACGGTGAAAACCTGGCCTATTTCCCTAAAGGGTTTATTGAGAATATGTTTTTCGTCT<br>CAGCCAATCCCTGGGTGAGTTTCACCAGTTTTGATTTAAACGTGGCCAATATGGACAACTTC<br>TTCGCCCCCGTTTTCACCATGGGCAAATATTATACGCAAGGCGACAAGGTGCTGATGCCGCT<br>GGCGATTCAGGTTCATCATGCCGTTTGTGATGGCTTCCATGTCGGCAGAATGCTTAATGAATT<br>ACAACAGTACTGCGATGAGTGGCAGGGCGGGGCGTAA
P_{tuf}	TACCGCTGACTGGGTTCTGGCTCCAAAGTGGCAAGAAACCCAGAAACCCAGTACAATGTA<br>GCGAGTGTCTTGTGCCGCAAGCGCAAGCCAACTACACGAGACGTCCAGGAGGACAATTAC
Signal peptide	ATGAAATACTTTAGGCGCATATTGAGCAGTATTATTGCAATCTTTTTGTGCATGTCAGCCTTC<br>ACGGGCACCGCAACGGCC
Supermutant IL2 gene	GCGCCCACGAGCTCGTCCACGAAGAAGACGCAACTCCAACTGGAACATCTGCTGCTCGAT<br>CTGCAGATGATTCTCAATGGAATTAATAACTACAAAAACCCCAAGCTGACCCGGATGCTCAC<br>GGCGAAGTTTACATGCCCAAGAAGGCCACCGAGCTCAAACATCTGCAGTGTCTCGAAGA<br>AGAGCTCAAACCCCTGGAAGAGGTTCTCAACCTCGCCCAATCCAAGAACTTCCATTTTGAC<br>CCGCGCGATGTGGTGTCCAACATTAACGTGTTCGTCCTGGAGCTGAAGGGATCCGAGACCA<br>CGTTCATGTGCGAGTATGCTGACGAGACCGCTACCATCGTCGAGTTCCTCAACCGTTGGATT<br>ACCTTTTGTCAAAGCATCATTTCGACCCTGACCTAA
Nanoluc gene	ATGGTTTTTACTCTGGAAGATTTTGTTGGCGATTGGCGTCAGACCGCGGGTTATAATTTGGAT<br>CAAGTCCTGGAACAGGGTGGCGTAAGCTCTCTGTTCCAGAACCTGGGTGTGAGCGTGACG<br>CCGATTCAGCGCATCGTTCTGTCCGGCGAGAACGGTCTGAAAATTGATATTCATGTGATCAT<br>CCCGTACGAAGGCCTGAGCGGTGACCAAATGGGTCAAATCGAGAAAATCTTTAAAGTCGTC<br>TACCCAGTTGACGATCACCACTTCAAGGTTATCTTGCATTACGGTACGCTGGTGATTGATGG

	TGTGACCCCGAATATGATTGACTATTTCGGCCGTCCGTATGAAGGCATTGCTGTTTTTGACGG<br>TAAAAAGATCACCGTCACCGGTACCCTGTGGAATGGCAATAAGATTATTGACGAGCGTCTG<br>ATTAACCCGGACGGCAGCCTGCTGTTCCGCGTGACCATCAACGGTGTCACGGGTTGGCGTC<br>TGTGCGAGCGCATCCTGGCATAA

Table S1. DNA sequence of plasmid components used in this paper. The underlined ATG represents where the translation starts.
